# Photo-electrochemical activation of persulfate for the simultaneous degradation of microplastics and personal care products†

**DOI:** 10.1039/d4ra01449a

**Published:** 2024-05-20

**Authors:** Jiacheng Huang, Wanyue Wang, Tao Wu, Xin Ren, Xuesong Zhao

**Affiliations:** a Key Laboratory of Environmental Materials and Pollution Control, Education Department of Jilin Province Siping 136000 China renxin@jlnu.edu.cn zhaoxuesong008@163.com; b College of Engineering, Jilin Normal University Haifeng Street, Tiexi Dist Siping 136000 China

## Abstract

The recent widespread use of microplastics (MPs), especially in pharmaceuticals and personal care products (PPCPs), has caused significant water pollution. This study presents a UV/electrically co-facilitated activated persulfate (PS) system to co-degrade a typical microplastic polyvinyl chloride (PVC) and an organic sunscreen *p*-aminobenzoic acid (PABA). We investigated the effect of various reaction conditions on the degradation. PVC and PABA degradation was 37% and 99.22%, respectively. Furthermore, we observed alterations in the surface topography and chemical characteristics of PVC throughout degradation. The possible degradation pathways of PVC and PABA were proposed by analyzing the intermediate products and the free radicals generated. This study reveals the co-promoting effect of multiple mechanisms in the activation by ultraviolet light and electricity.

## Abstract 2

The recent widespread use of microplastics (MPs), especially in pharmaceuticals and personal care products (PPCPs), has caused significant water pollution.

## Graphical abstract

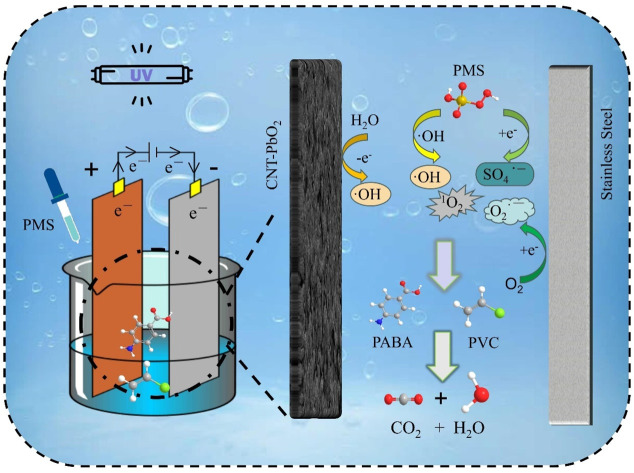

## 1 Introduction

It is becoming a widespread concern that misusing pharmaceutical and personal care products (PPCPs) can harm the ecology.^1^ PPCPs include medical drugs and household products such as antibiotics, sunscreen, toothpaste, and disinfectants.^2^ Most refractory PPCPs eventually enter various water bodies through sewage plants, threatening the ecological safety of the aqueous environment.^3^ In addition, PPCPs are pseudo-persistent in water; thus, they are frequently detected worldwide.^4,5^ Therefore, potential environmental threats from PPCPs are receiving extensive attention.

Plastics have become more frequently used in recent decades, severely polluting the environment.^6^ They are difficult to biodegrade and can exist in the ecological environment for hundreds of years. However, combined physical, chemical, and biological environmental processes can decompose them into smaller particles; those smaller than 5 nm are called microplastics (MPs).^7^

Polyvinyl chloride (PVC) is a popular plastic used globally in construction, medical, and packaging.^8^ Currently, PVC wastes are disposed of *via* landfilling, incineration, and recycling.^9^ Although landfilling is effective, its choice has significantly reduced.^10^ Landfills release additives and harmful gases that pollute groundwater and soil.^9,11^ Besides being another effective disposal method of plastic waste, incineration generates energy. However, it releases high amounts of CO_2_ and highly toxic dioxins.^12^ Chemical recycling is considered a promising strategy for recycling and reuse.^13^ On the other hand, plastic recycling can reduce pollution from landfills and oceans and reduce harmful emissions.^14^ Therefore, finding a technology to degrade PPCPs and PVC MPs effectively is necessary.

Consequently, a plethora of water treatment technologies have been developed, including membrane filtration technology, photocatalysis, encompassing adsorption, microwave catalysis, and other advanced methodologies. The membrane filtration technology exhibits remarkable adaptability and simplicity in operation; however, the persistent issue of membrane fouling poses a formidable challenge to overcome. On the other hand, photocatalysis represents an environmentally friendly and practical approach. Wei *et al.*^15^ successfully synthesized the MIL-88B(Fe) catalyst with diverse morphologies and achieved efficient tetracycline removal in wastewater. Nevertheless, the suspension of catalysts in water impedes their recovery and may result in a certain degree of secondary contamination. The adsorption technology exhibits the characteristics of facile operation, low energy consumption, and high efficiency. Bi *et al.*^16^ employed fluorination modification to synthesize UiO-67 materials with pronounced hydrophobicity and exceptional water stability, thereby achieving the efficient removal of volatile organic compounds (VOCs) in humid environments. However, adsorption technology merely facilitates the transfer of pollutants without undergoing fundamental degradation.^17^ The microwave catalytic method offers the advantages of operating under mild conditions, conserving energy, and preserving the environment. Consequently, many researchers have used microwaves to enhance pollutant degradation. Wang *et al.*^18^ developed a highly efficient microwave-induced catalyst using the high-temperature carbonization method, presenting a novel approach for environmental remediation. However, the utilization of microwave catalysis is subject to certain limitations and may not possess universal applicability across all chemical reactions. The advanced oxidation process is a crucial approach to achieving the environment-friendly disposal of organic pollutants in water. The use of a single advanced oxidation processes (AOP) is associated with drawbacks such as prolonged reaction time, limited efficiency, and narrow applicability.^19^ Therefore, the combined utilization of multiple AOPs has garnered considerable attention due to their potential for synergistic effects and improved problem-solving capabilities.^20^

Activated persulfate and electrochemical oxidation technologies are advanced oxidation processes effective in removing contaminants from water. The sulfate radical (SO_4_˙^−^, *E*_0_ = 3.1 V *vs.* NHE) promotes polyethylene microplastic degradation.^21^ Electrochemical oxidation destroys and mineralizes organic compounds using the hydroxyl radical (˙OH, *E*_0_ = 2.7 V *vs.* NHE) that generates high redox potentials.^22^ This method has attracted attention because of its simplicity, the absence of secondary pollution, and its high mineralization degree.^23,24^

The anode material is crucial for determining the degree of pollutant mineralization and degradation efficiency.^25^ Therefore, researchers have explored various anode materials, such as BDD,^26^ RuO_2_,^27^ SnO_2_,^28^ and PbO_2_,^29^ owing to their high conductivity, excellent stability, and low cost.^30^ The PbO_2_ electrode has been investigated for degrading organics in wastewater by modifying it to improve the degradation efficiency and electrocatalytic activity.

Carbon nanotubes (CNT) have excellent catalytic activity due to their relatively large surface area and high conductivity. Therefore, they are widely used in electrode doping.^31^ For example, Duan *et al.*^32^ prepared the CNT-PbO_2_ electrode by thermal- and electro-deposition, improving its catalytic activity toward PbO_2_. Elsewhere, You *et al.*^33^ doped CNT through an oxygen bubble template method to improve its organic matter removal efficiency in water. The results show that CNT doping can effectively improve the electrocatalytic activity of the PbO_2_ electrode.

In this study, we prepared CNT-PbO_2_ electrodes by electro-deposition. Also, a UV/electric co-activated PS system was constructed to achieve the co-removal of MPs with PABA, revealing the co-promoting effects of multiple mechanisms in PS activation by UV (20 W, *λ*_¼_ 365 nm) and electricity. Using scanning electron microscopy (SEM), electron dispersive spectroscopy (EDS), and X-ray diffractometry (XRD), we examined the morphology, crystallinity, and element composition of the electrodes. The degradation performance of the UV/electric co-activated persulfate (PMS) system was evaluated with simulated wastewater and landfill leachate effluent as the research object.

## 2 Experiment

### 2.1 Materials

Titanium plate (titanium, 99.9%) and multi-walled carbon nanotubes (MWCNT) were purchased from Shenzhen Suiheng Technology Co., China. Sodium fluoride, oxalic acid, nitric acid, anhydrous sodium carbonate, lead nitrate, potassium persulfate, lead oxide, tin tetrachloride, antimony trichloride, sodium hydroxide, isopropanol, anhydrous ethanol, and acetone were procured from China National Pharmaceutical Chemical Reagent Co. All chemicals were analytically pure and did not require further purification. All solutions were prepared with deionized water.

### 2.2 Preparation of electrodes

The titanium sheets were repeatedly polished using sandpapers with varied roughness (120, 240, 400, and 600 grit). Then, the sheets were sequentially ultrasonically rinsed with acetone, ethanol, and distilled water for 15 min each. Subsequently, the polished titanium sheets were etched with oxalic acid (20%) at 100 °C for 4 h until a rough interface was obtained. It was then stored in 1% oxalic acid.

The SnO_2_–Sb base layer was prepared by thermal deposition. The SnO_2_–Sb solution contained 100 g SnCl_4_, 10 g SbCl_3_, 66 mL concentrated hydrochloric acid, and 200 mL isopropanol, dissolved in a 500 mL volumetric flask using isopropanol fixation. The solution was entirely devoid of water. The previously stored electrode sheet was removed, and deionized water was used to remove the oxalic acid solution on the surface. The SnO_2_–Sb solution was evenly dripped onto the electrode surface and dried at 120 °C for 10 min. After that, it was placed in a muffle furnace at 550 °C for 10 min. This procedure was repeated 10–12 times, with the last round lasting 1 hour. The titanium plate was placed in a plating solution consisting of 0.1 M PbO and 3.5 M NaOH. A 3 mA cm^−2^ current density was applied to the bottom layer for 60 min at 40 °C to obtain the α-PbO_2_ layer. Finally, the β-PbO_2_ active layer was obtained by electro-deposition in 100 mL of an acidic solution consisting of 0.5 mol L^−1^ Pb(NO_3_)_2_, 1 mol L^−1^ HNO_3_, and 0.5 mol NaF at a 15 mA cm^−2^ current density and 65 °C for 60 min. In preparing CNT-modified electrodes, CNT was added to the acidic solution.

### 2.3 Electrode characterization

The morphology and elemental composition of the electrodes were examined using a scanning electron microscope (SEM) model JSM-7900F equipped with energy-dispersive X-ray spectrometer (EDS). The sample was also characterized by an X-ray diffractometer (PANalytical/Empyrean 2). Further analyses with linear sweep voltammetry (LSV), cyclic voltammetry (CV), and electrochemical impedance spectroscopy (EIS) were done using a conventional three-electrode electrochemical workstation (CHI760E). The prepared electrode works as an electrode, the platinum electrode as the opposite electrode, and the saturated calomel electrode as the reference electrode. The electrochemical test was done at ambient temperature.

### 2.4 Electrochemical experiments

In the photoelectric coupling system, the CNT-modified PbO_2_ electrode served as the anode, while the stainless steel plate was the cathode. The electrochemical degradation was carried out by adding 10 mg PVC to 200 mL of electrolyte. The anode and cathode were 2 cm apart and parallel to each other. The UV lamp was 10 cm away from the degradation liquid surface during the process. The influence of various conditions (electrolyte concentration, initial pH, temperature, PMS dosage, and current density) on the degradation was determined. The electrolyte was composed of 10 mg L^−1^ PABA and varied electrolyte concentrations.

Two groups of degradation experiments were set up under the same conditions. A set of degradation experiments lasted 1 hour, with samples taken at regular intervals to measure the PABA concentration. The other group of experiments lasted 8 hours. After the experiment, the degradation solution was extracted, filtered, dried, and weighed to estimate PVC degradation.

## 3 Results and discussion

### 3.1 Structure and morphology

#### 3.1.1 SEM and EDS analyze

**Fig. 1:**
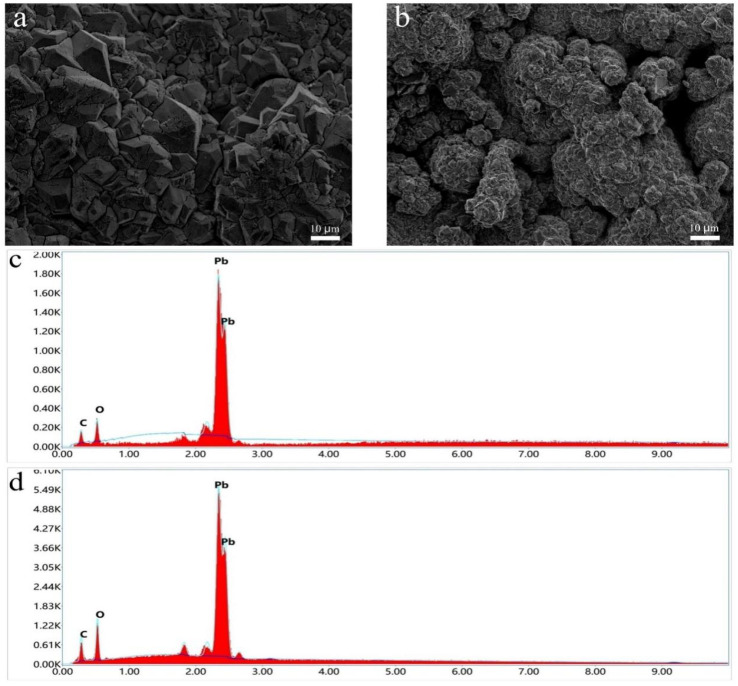
SEM images of (a) PbO_2_ and (b) CNT-PbO_2_ electrodes. Energy spectra of (c) PbO_2_ and (d) CNT-PbO_2_ electrodes.

Fig. 1a and b are the SEM images of PbO_2_ and CNT-PbO_2_ electrodes. They show pyramidal structures and the surface morphology changes arising from the CNT doping of the electrodes. The CNT-PbO_2_ electrode surface was more porous than that of the PbO_2_ electrode. Therefore, the CNT-PbO_2_ electrode exhibits a significantly enhanced specific surface area, which is further supported by the characterization results presented in Table 1. The enhancement of catalytic oxidation capacity is well recognized to be facilitated by a high specific surface area. The CNT doping reduced the particle size of the electrode as the connection between the crystals became closer. The validity of this perspective is further substantiated by the outcomes obtained from the subsequent grain size calculation.

**Table 1:** PbO_2_ and CNT-PbO_2_ BET specific surface area test data

Electrode	PbO_2_	CNT-PbO_2_
BET specific surface area	20.7275 ± 0.2202 m^2^ g^−1^	108.4096 ± 0.5335 m^2^ g^−1^

The EDS elemental analysis showed that the carbon content of PbO_2_ and CNT-PbO_2_ electrodes was 0.01% and 3.59%, respectively (Fig. 1c and d). These results also proved that CNT was successfully doped onto the PbO_2_ film.

#### 3.1.2 XRD analyze

**Fig. 2:**
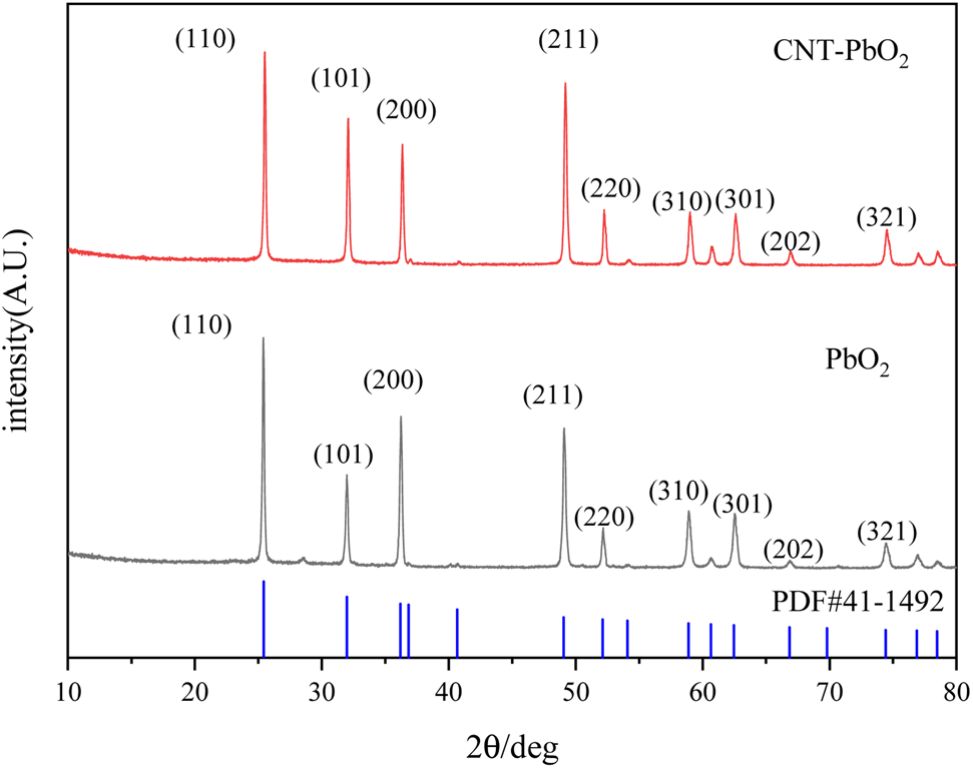
XRD patterns of PbO_2_ and CNT-PbO_2_ electrodes.

The XRD examination of the materials' crystallinity showed similar diffraction peaks for both electrodes (Fig. 2). Their diffraction peaks also matched that of the β-PbO_2_ standard card (PDF#41-1492), indicating accurate attribution. The 2*θ* of the XRD pattern was 25.4°, 31.9°, 36.1°, 49.1°, 52.1°, 58.8°, 62.5°, 66.8°, and 74.4°, corresponding to the diffraction peaks of (110), (101), (220), (211), (220), (310), (201), (202), and (321) crystal plane reflections, respectively. The diffraction peak intensity of the (101) and (211) crystal faces of the CNT-PbO_2_ electrode was higher than that for the PbO_2_ electrode. This difference proves that CNT doping enhanced the β-PbO_2_ growth on the (101) and (211) crystal planes. No diffraction peak corresponding to CNT was detected because the CNT content in the β-PbO_2_ film was below the XRD detection limit.^33^ Further, using Scherrer's formula (eqn (1)), we obtained the grain size of the electrodes.1
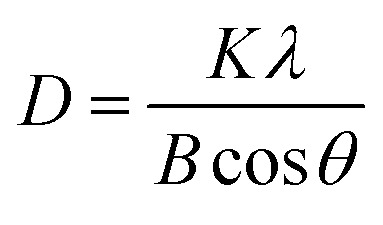


The grain size of the CNT-PbO_2_ electrode was 35.19 nm, smaller than that of the PbO_2_ (37.73 nm) electrode. Therefore, the CNT doping lowered the grain size, and the specific surface area and active site were increased, thereby facilitating the degradation of organic pollutants.^34^

#### 3.1.3 XPS analysis

**Fig. 3:**
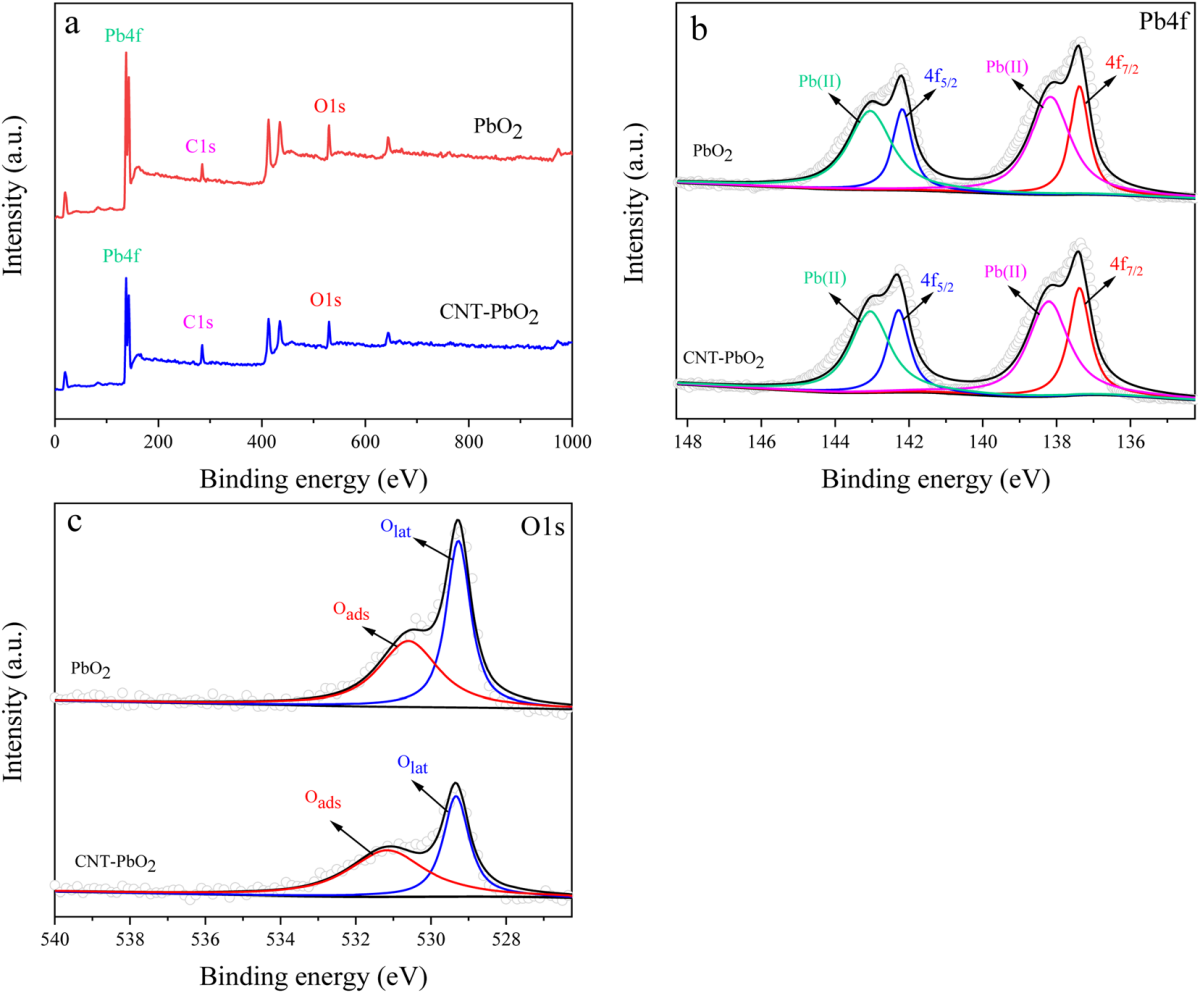
XPS spectra of PbO_2_ and CNT-PbO_2_ electrodes (a) total spectra (b) Pb4f (c) O 1s.

XPS examined the chemical functionalities on the PbO_2_ and CNT-PbO_2_ electrodes. Fig. 3b is the XPS spectrum of Pb 4f, confirming the presence of Pb(ii) and Pb(iv). Both electrodes have 137.35 and 142.25 eV peaks corresponding to Pb4f_7/2_ and Pb4f_5/2_, respectively.^35^ Moreover, the binding energy difference between the peaks belonging to Pb(iv) was about 4.9 eV, proving that β-PbO_2_ films were formed on both the electrodes.^36^Fig. 3c shows the XPS characteristic peaks of O near 529 and 531 eV. As the binding energy increases, these peaks correspond to lattice oxygen (O_lat_) and adsorbed oxygen (O_ads_), respectively.^37^ The relative content of the oxygen species (Table 2) was calculated. Here, the O_ads_ content of the PbO_2_ electrode was 47.97%, lower than that of the CNT-PbO_2_ electrode (56.14%). O_ads_ is crucial for electrochemical catalysis, and the higher the O_ads_ content, the more efficient the pollutant mineralization.^38^ Therefore, it can be inferred that CNT doping can improve the catalytic oxidation ability of the PbO_2_ electrode.

**Table 2:** XPS data of the oxygen (O) functionalities on the electrodes

Electrode	Binding energy/eV		O_ads_ content/%	O_lat_ content/%
	O_ads_	O_lat_		
PbO_2_	530.6	529.25	47.97	52.03
CNT-PbO_2_	531.15	529.3	56.14	43.86

### 3.2 Electrochemical measurements

#### 3.2.1 Linear sweep voltammetry

**Fig. 4:**
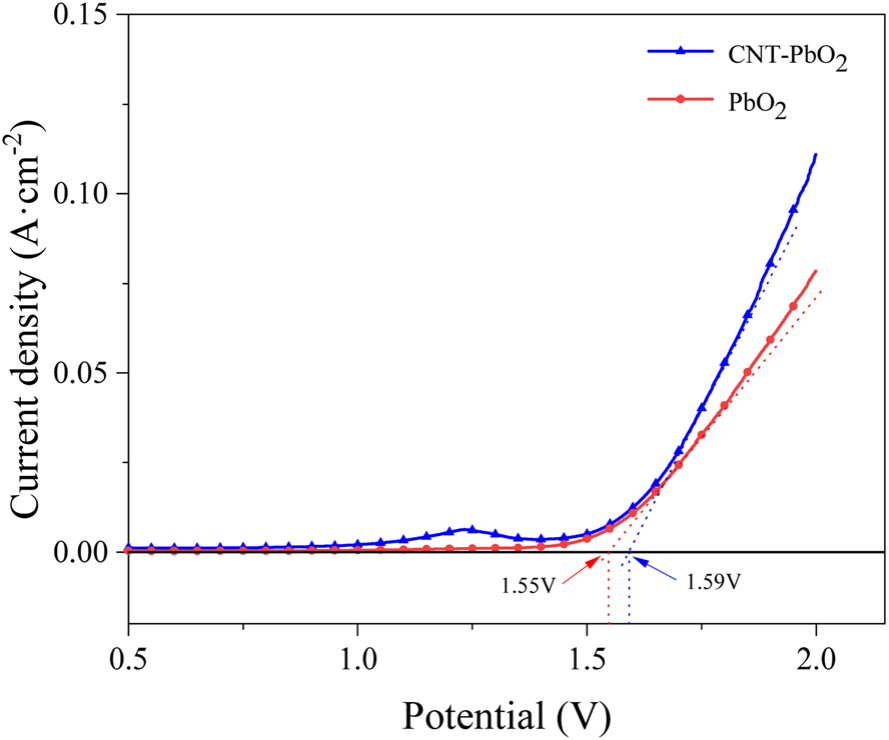
Linear scanning voltammetric curves for PbO_2_ and CNT-PbO_2_ electrodes.

Linear sweep voltammograms were measured in 0.5 mol L^−1^ NaSO_4_ solution at a 5 mV s^−1^ sweep rate (Fig. 4). The oxygen evolution potential of PbO_2_ and CNT-PbO_2_ electrodes was 1.55 and 1.59 V, respectively. The high oxygen evolution potential of the CNT-PbO_2_ electrode indicates that CNT doping improves the oxygen evolution capacity of the anode. Oxygen evolution potential is essential in measuring the electrochemical activity of anodes.^39^ A high oxygen evolution potential can increase the electrochemical activity and promote degradation reactions.^35,40^ Therefore, the CNT-PbO_2_ electrode may have superior electrocatalytic ability than the PbO_2_ electrode.

#### 3.2.2 Cyclic voltammetry

**Fig. 5:**
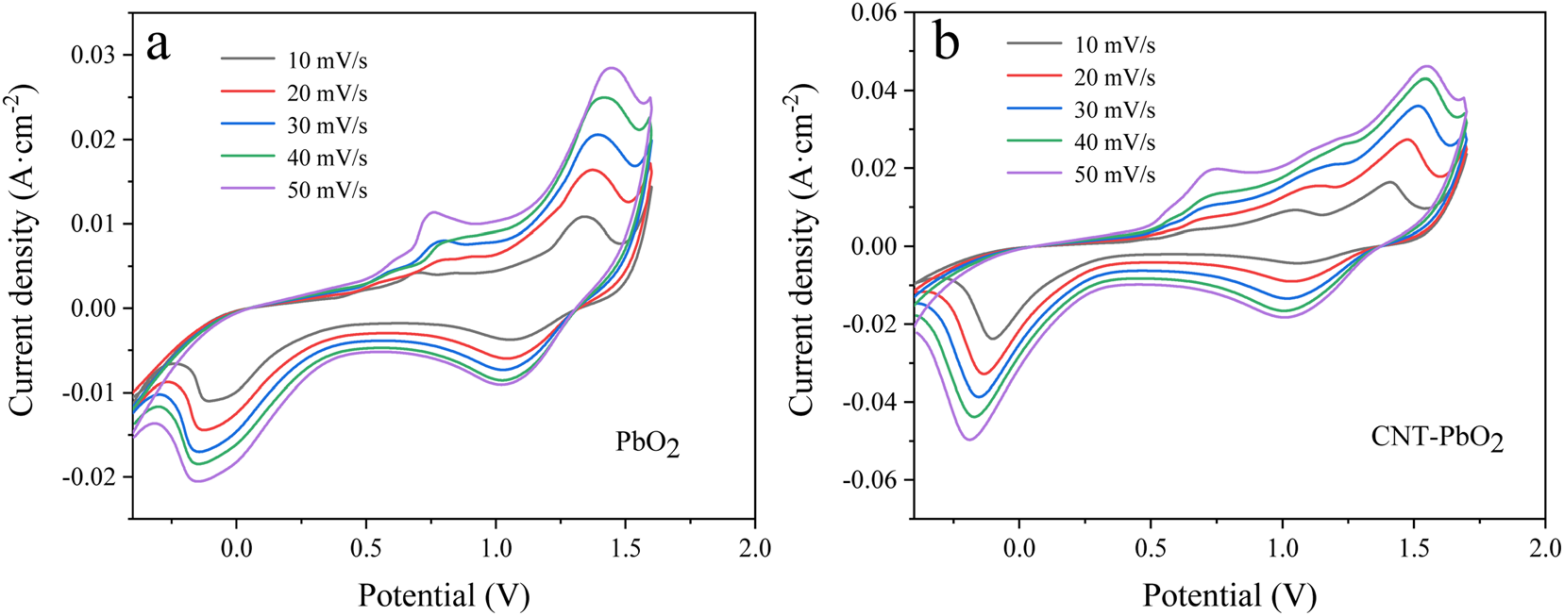
Cyclic voltammetry curves measured at different sweep speeds (a) PbO_2_ (b) CNT-PbO_2_.

**Fig. 6:**
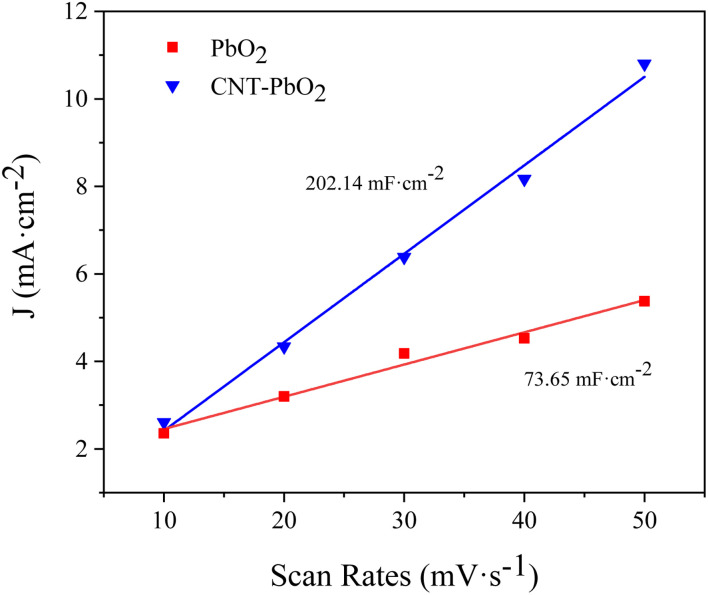
Electrochemical surface area spectrum of PbO_2_ and CNT-PbO_2_ electrodes.

Fig. 5a and b are the CV spectra of PbO_2_ and CNT-PbO_2_ electrodes. Both electrodes have oxidation and reduction peaks representing the REDOX of Pb(ii) and Pb(iv).^41^Fig. 6 depicts the electrochemical surface area (ECSA) spectrum of the two electrodes. The active surface area of the CNT-doped electrode (220.14 mF cm^−2^) is much larger than that of the pure PbO_2_ electrode (73.65 mF cm^−2^). Therefore, CNT doping increases the active specific surface area, which is advantageous for enhancing the electrode's electrocatalytic oxidation capability. In summary, it can be inferred that the CNT-doped electrode had a higher electrochemical catalytic activity and stronger degradation ability of organic pollutants than the undoped electrode.

#### 3.2.3 Electrochemical impedance spectroscopy

**Fig. 7:**
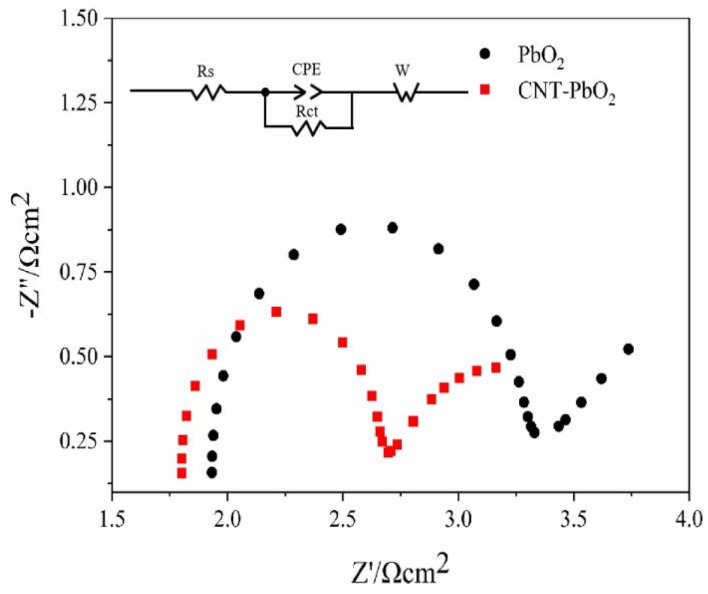
Electrochemical impedance and equivalent circuit of PbO_2_ and CNT-PbO_2_ electrodes.

The EIS test characterized the two anodes in a 0.5 mol L^−1^ Na_2_SO_4_ solution. Fig. 7 shows the EIS fitting and equivalent circuit diagram of the electrode. We observed that the capacitive reactance arc of the electrodes was a regular semicircle, suggesting that their reaction was similar. Therefore, the same equivalent circuit diagram was used to fit the data. Typically, the diameter of the semicircle in the EIS diagram represents the charge transfer resistance (*R*_ct_). Therefore, the smaller the diameter, the smaller the *R*_ct_ and the lesser the resistance to the reaction.^42^ Since the starting point of the semicircle was not 0, the solution resistance (*R*_s_) was considered when calculating the *R*_ct_. The estimated *R*_ct_ value for the PbO_2_ and CNT-PbO_2_ electrodes was 1.40 Ω and 0.89 Ω, respectively. Moreover, in comparison to the multi-layer CNT-PbO_2_ anode prepared by Xia *et al.*^43^ and the three-dimensional porous PbO_2_-CNTs composite electrode prepared by You *et al.*,^33^ the CNT-PbO_2_ anode fabricated in this study exhibits a lower charge transfer resistance, thereby suggesting its superior electrochemical activity and charge transfer capability. The results show that CNT doping increased the charge transfer ability of the electrode.

### 3.3 Morphology characterization of PVC

**Fig. 8:**
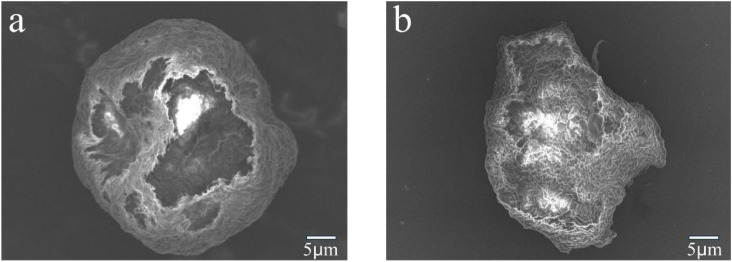
SEM images of (a) untreated PVC and (b) treated PVC.

Fig. 8a and b depict the SEM surface morphology of the PVC before and after an 8 h degradation, respectively. Before treatment, PVC evinced a more regular circle; the surface was smoother and more uniform, with no rupture traces. When PVC was degraded for 8 h, the regular circle was destroyed entirely, with cracks and holes appearing on the surface, yielding a granular shape. Moreover, because the treated PVC's surface was decomposed, the exposure range of the internal PVC became larger (Fig. 8b). These changes indicated that PVC could have been oxidized and dechlorinated during the degradation.

### 3.4 PVC characterization

**Fig. 9:**
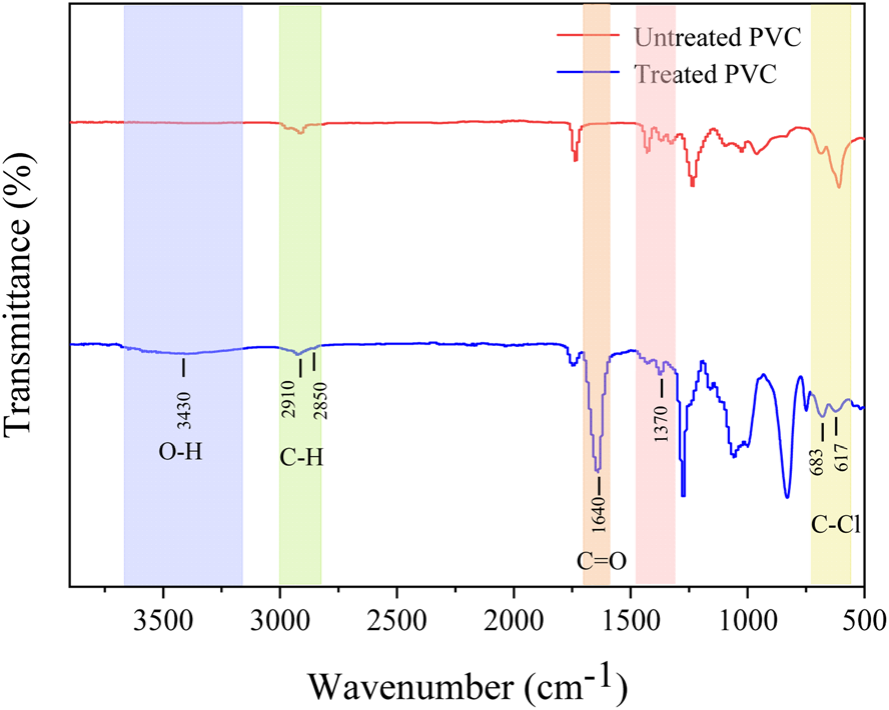
FTIR spectra of untreated PVC and treated PVC.

FTIR examined the PVC's functional groups to reveal any changes in its chemical properties after the photoelectric coupling system degradation. The absorption bands at 1370, 2910, and 2850 cm^−1^ (Fig. 9) belong to the C–H groups. After the reaction, the characteristic peak of the C–H group was weakened, suggesting that oxidation had occurred, removing some C–H bonds.^44^ After the reaction, a C=O group absorption band at 1640 cm^−1^ was formed. It ensued because the free radical chain centered on the C atom formed by PVC during the oxidation was oxidized to oxygen-containing functional groups.^45^ Distinct absorption bands characteristic of the C–Cl bond were observed at 673 and 617 cm^−1^. However, their strength decreased significantly after the treatment, confirming that oxidation and dechlorination occurred during PVC degradation. An absorption band near 3430 cm^−1^ belonging to the O–H group appeared after the degradation, confirming that the degradation of PVC was accompanied by oxidation.

### 3.5 Individual degradation of PVC and PABA

Fig. S1–S10† illustrate the data obtained from investigating the effect of UV/electric co-activated persulfate system on PVC and PABA degradation under varied reaction conditions.

### 3.6 The analysis of degradation

#### 3.6.1 Analysis of different systems

**Fig. 10:**
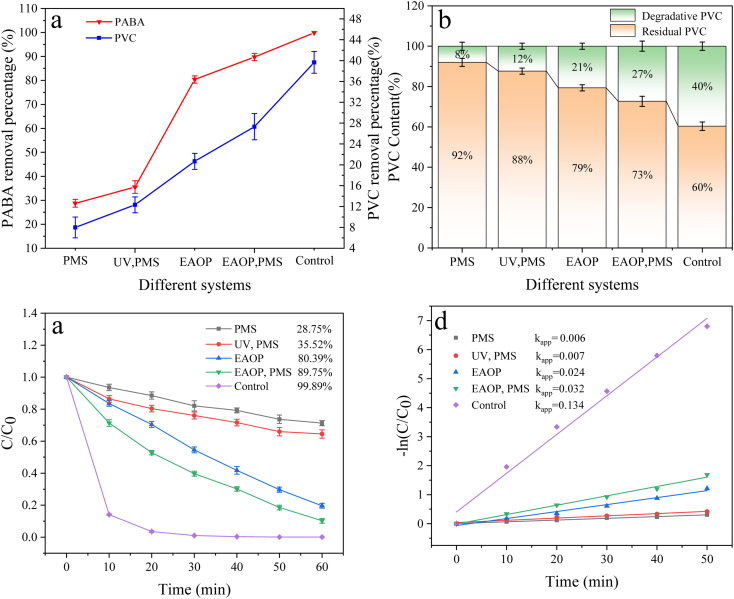
(a) Effect of different systems of PVC and PABA removal percentage. (b) Effect of different systems on PVC degradation. (c) Effect of different systems on PABA degradation. (d) First-order kinetic model of PABA degradation.

Fig. 10 illustrates the degradation rates of PVC and PABA in different reaction systems. The single PMS system exhibits the poorest degradation effect on PVC and PABA, with degradation rates of 8% and 28.75%, respectively. Furthermore, the activation effect of ultraviolet light on PMS is significantly inferior to that of electrocatalytic activation due to the latter's inherent oxidation technology for sewage treatment. Therefore, the following focus will be directed towards electrocatalysis. The doping amount of CNT was also investigated in this study, and the results demonstrate that a doping concentration of 2 g L^−1^ leads to enhanced removal efficiency for PABA and PVC in the reaction system. It is noteworthy that excessive CNT loading (3 g L^−1^) weakens the pollutant removal performance, possibly due to agglomeration issues hindering the effective incorporation of CNT into the β-PbO_2_ film, thereby impacting the electrocatalytic activity of the anode material. Detailed data are presented in Fig. S11 and S12.†

#### 3.6.2 Effect of PMS dosage

**Fig. 11:**
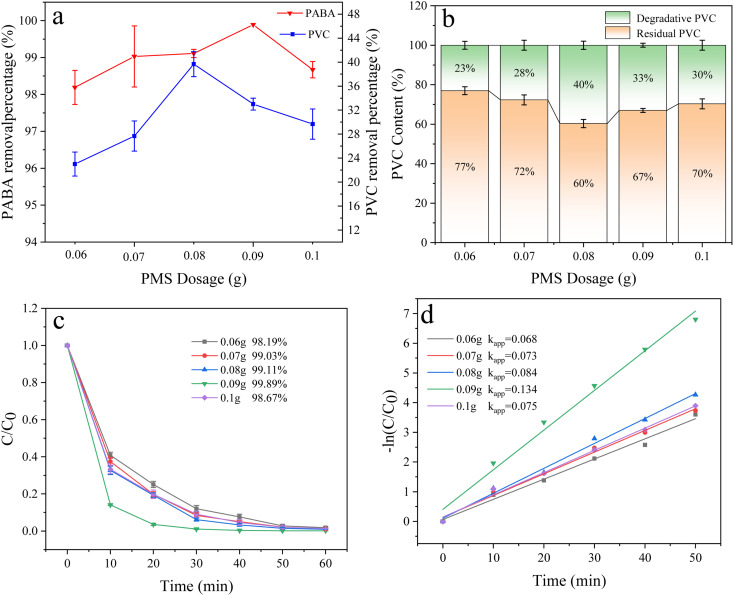
(a) Effect of PMS dosage on PVC and PABA removal percentage. (b) Effect of PMS dosage on PVC degradation. (c) Effect of PMS dosage on PABA degradation. (d) Shows the first-order kinetic model of PABA degradation as the PMS amount changed.

In the experiment, the PMS dosage can influence the production of SO_4_˙^−^ and ˙OH. Herein, the effect of PMS on the degradation of PABA and PVC was investigated (Fig. 11) by setting PMS dosage as 60, 70, 80, 90, and 100 mg. As the PMS dosage increased, the degradation of PABA and PVC increased initially before decreasing. The optimal amount of PMS for PABA and PVC degradation was 90 and 80 mg, respectively (Fig. 11b and c). Fig. 11d shows the first-order kinetic model of PABA degradation as the PMS amount changed. We observed that the reaction kinetic constant was largest when the PMS dosage was 90 mg. The reaction's first-order kinetics and degradation trend showed that the degradation efficiency improved with the PMS amount. However, excessive PMS addition would inhibit the process. This is attributed to the potential quenching of excessive PMS by SO_4_˙^−^ and ˙OH, resulting in the formation of weakly oxidizing SO_5_˙^−^ species that impede pollutant degradation.^46^ On the other hand, SO_4_˙^−^ was produced from the breaking of the O–O bond in PMS, and the SO_4_˙^−^ in the system was proportional to PMS dosage. Excessively large PMS dosage inhibited SO_4_˙^−^ formation by generating S_2_O_8_˙^−^, thereby lowering the degradation effect of PABA and PVC.

#### 3.6.3 Effect of temperature

**Fig. 12:**
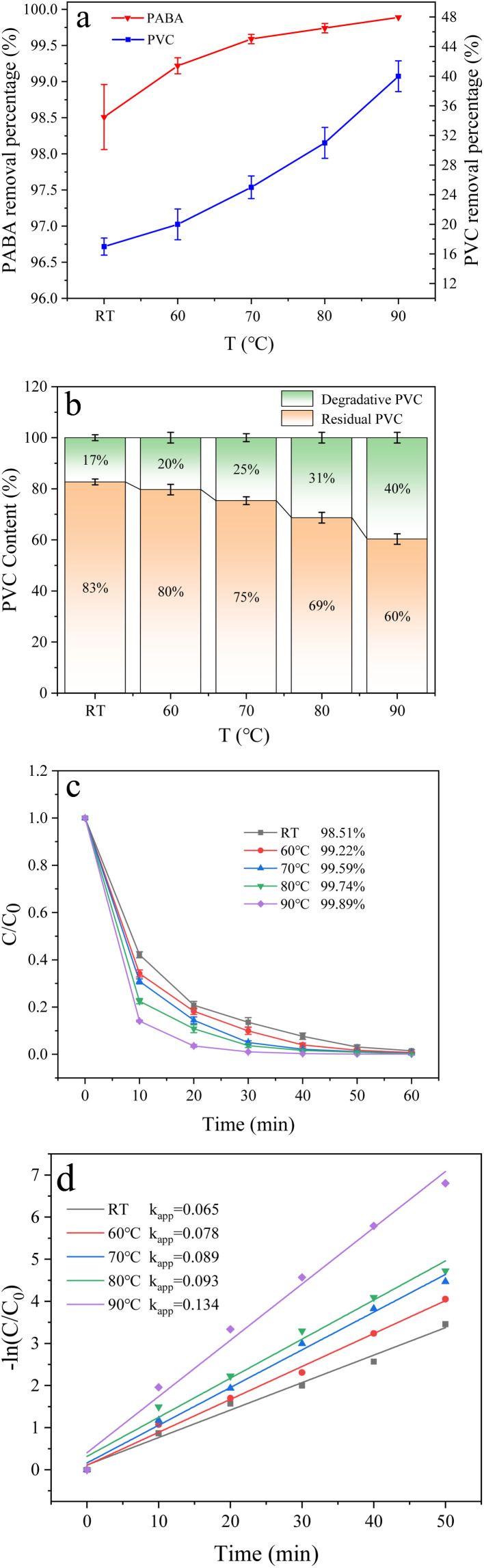
(a) Effect of temperature on PVC and PABA removal percentage. (b) Effect of temperature on PVC degradation. (c) Effect of temperature on PABA degradation. (d) First-order kinetic model of PABA degradation.

Temperature crucially affects the degradation efficiency. The results show that increasing the reaction temperature can improve the dechlorination of chlorine-containing compounds.^47^ Therefore, we investigated the effect of temperature change on PABA and PVC degradation at room temperature, 60 °C, 70 °C, 80 °C, and 90 °C (Fig. 12a). We noticed that the removal of both pollutants increased with temperature. Fig. 12b illustrates the influence of temperature on PVC degradation when co-degrading the two pollutants at various temperatures. Here, the PVC removal rate increased with temperature, optimized at 90 °C at 40%. This limit may be because increasing the reaction temperature affects the PVC structure's stability. Higher temperatures would denature the unstable structures and aggravate the C–Cl bond fracture.

Similarly, Fig. 12c shows the effect of temperature change on PABA degradation when co-degrading the pollutants. The first-order kinetic curves of PABA at varied temperatures are shown in Fig. 12d. PABA's removal and reaction rate increased with temperature. At 90 °C, the highest removal rate of PABA was achieved (Fig. 12c and d). The thermal activation of PMS is widely recognized as a common and effective method. Furthermore, an elevated reaction system temperature leads to a significant increase in the production of ˙OH through the reaction between sulfate radicals and water.^48^ Sun *et al.* observed that the thermal activation of persulfate can effectively degrade *p*-chloro-*m*-xylenol because the elevated temperature promotes the generation of more active substances from persulfate.^49^ Also, the temperature elevation facilitates the process of electron transfer within the solution, thereby augmenting the catalytic activity of PMS. Therefore, increasing the reaction temperature can promote the reaction and improve the degradation efficiency.

#### 3.6.4 Effect of current density

**Fig. 13:**
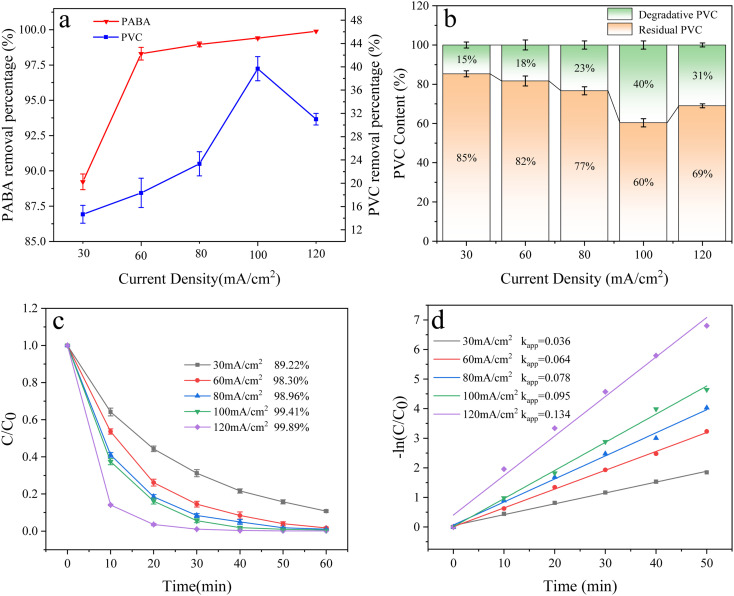
(a) Effect of the current density on PVC and PABA removal percentage. (b) Effect of current density on PVC degradation. (c) Effect of current density on PABA degradation. (d) First-order kinetic model of PABA degradation.

The electrocatalytic oxidation capacity depends on the current density in a reaction, which is decisive in degradation processes.^50^Fig. 13 illustrates the effect of current densities (30, 60, 80, 100, and 120 mA cm^−2^) on the degradation of PABA and PVC. PABA degradation increased with the current density. At 120 mA cm^−2^, the degradation reached 99.89% in 1 h (Fig. 13c). We fitted the experimental data with the first-order reaction kinetic model (Fig. 13d). We observed that the kinetic constant *k*_app_ of PABA degradation increased with the current density, probably on the corresponding increase in ˙OH production.^51^ The electron transfer rate will impact both the direct oxidation and indirect oxidation of the electrode, thereby accelerating the degradation rate.^52^ However, with the current density increase, the PVC degradation increased at first before decreasing. When the current density was 100 mA cm^−2^, the degradation reached 40%, but at 120 mA cm^−2^, it dropped to 31% (Fig. 13a and b). This drop occurred because a very high current density induces side reactions (such as oxygen and hydrogen evolution), which reduce effective current utilization and increase energy consumption. The Joule heating effect at the electrode interface may also contribute to this phenomenon.^53^ The excessive current density can cause higher electrode temperature, increasing the likelihood of H_2_O_2_ generation and reducing reactive oxygen species concentration in solution.^54^ Consequently, this diminishes the electrode's catalytic oxidation capacity.

#### 3.6.5 Effect of electrolyte concentration

**Fig. 14:**
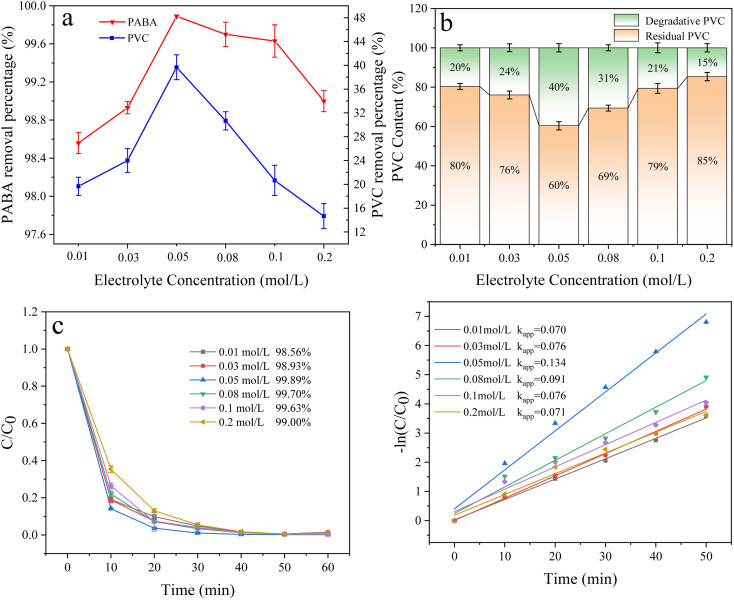
(a) Effect of electrolyte concentration on PVC and PABA removal percentage. (b) Effect of electrolyte concentration on PVC degradation. (c) Effect of electrolyte concentration on PABA degradation. (d) First-order kinetic model of PABA degradation.

The electrolyte reduces the reaction liquid's resistance and improves the solution's conductivity in electrocatalytic oxidation. This experiment investigated the effect of electrolyte concentration (0.01, 0.03, 0.05, 0.08, 0.1, and 0.2 mol L^−1^) on the degradation of PABA and PVC. As shown in Fig. 14a and c, PABA degradation increased with the electrolyte concentration. When the electrolyte concentration was 0.05 mol L^−1^, PABA removal reached 99.89%. However, when the concentration was increased, the removal efficiency declined. This phenomenon was also illustrated by the first-order kinetic fit for PABA degradation (Fig. 14d). Similarly, the PVC degradation increased before decreasing when the electrolyte concentration was varied. When the electrolyte concentration was 0.05 mol L^−1^, the PVC degradation reached 40% (Fig. 14b). Studies have shown that a very low electrolyte concentration affects the generation of active oxidizing substances. Still, a very high electrolyte concentration may lead to a short circuit,^55^ and the high concentration of electrolytes hinders the transfer of target pollutants to the electrode surface by facilitating the formation of a salt film,^56^ thus hindering organic matter removal. Increasing the electrolyte concentration would increase SO_4_^2−^ and oxidize it to S_2_O_8_^2−^ in the system. However, excess S_2_O_8_^2−^ would form H_2_O_2,_ which reacts with ˙OH and thus inhibits the degradation of organic matter. This phenomenon might have contributed to the degradation trend increasing and then decreasing.

2 2SO_4_^2−^ → S_2_O_8_^2^˙^−^ + 2e^−^

3 S_2_O_8_^2^˙^−^ + 2H_2_O → H_2_O_2_ + 2SO_4_^2−^ + 2H^+^

4 H_2_O_2_ → O_2_ + 2H^+^ + 2e^−^

5 H_2_O_2_ + 2˙OH → O_2_ + 2H_2_O

#### 3.6.6 Effect of pH

**Fig. 15:**
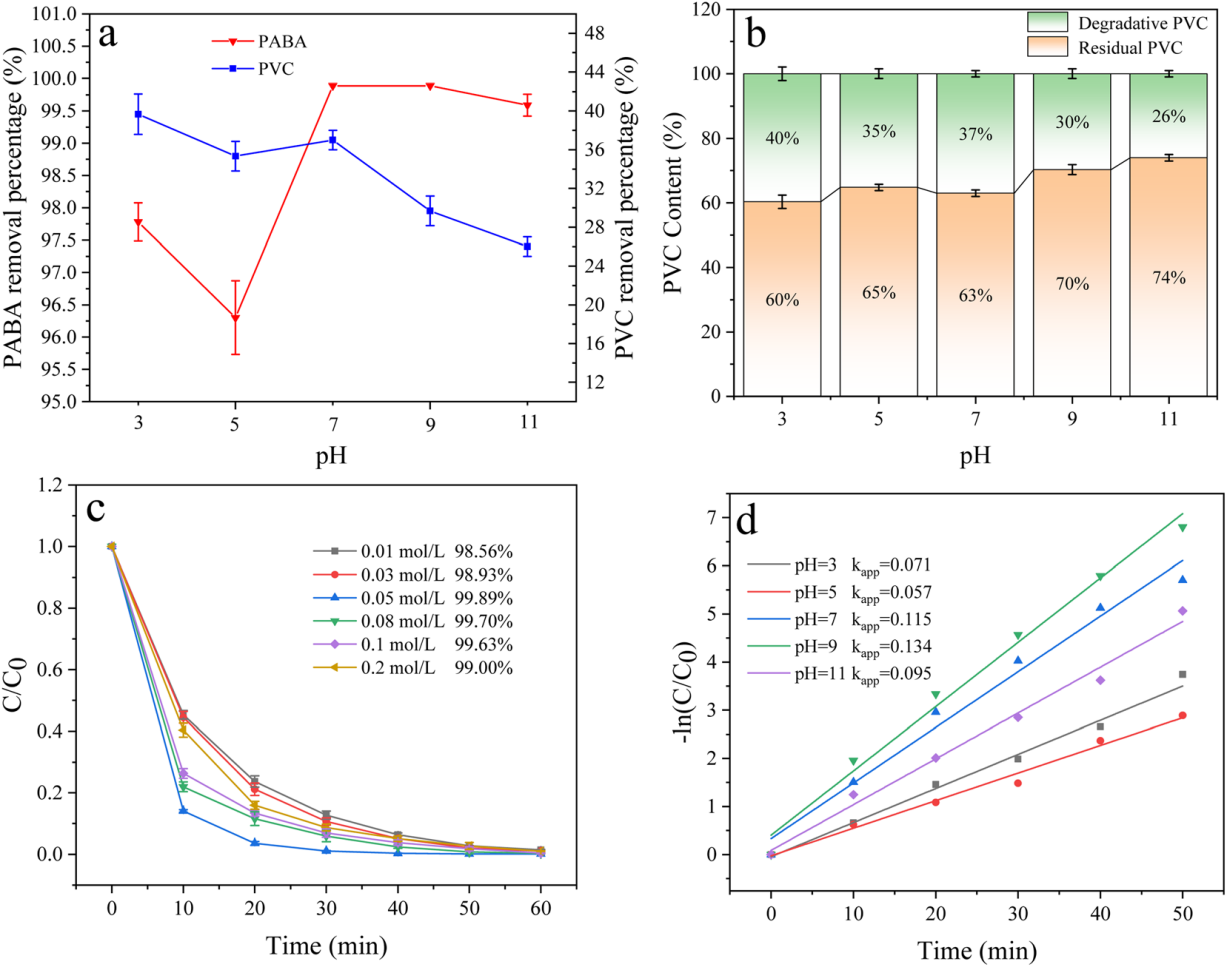
(a) Effect of pH on PVC and PABA removal percentage. (b) Effect of pH on PVC degradation. (c) Effect of pH on PABA degradation. (d) First-order kinetic model of PABA degradation.

In electrocatalytic oxidation, the pH value affects the generation of active oxidizing substances and thus, the degradation efficiency of organic matter. In this experimental system, pH mainly affected indirect oxidation. Therefore, we studied the effect of varied pH (3, 5, 7, 9, and 11) on PABA and PVC degradation (Fig. 15c). PABA degradation was the fastest and most efficient at pH 9, whose rate constant *k*_app_ was the highest. Fig. 15d shows the first-order reaction kinetics of pH effect on PABA degradation. Under alkaline conditions, the electrocatalytic process primarily involves an indirect reaction. Generally, ˙OH is more readily generated and exhibits a longer lifespan under alkaline conditions,^57^ thereby resulting in a higher concentration of ˙OH in the solution at pH = 9. This enhanced presence of ˙OH is conducive to efficient pollutant removal. However, it should be noted that excessive alkalinity can lead to oxygen evolution at the anode, consequently reducing current efficiency. As shown in Fig. 15b, the highest PVC removal (40%) was attained at pH 3 because Cl^−^ could easily undergo redox reactions to form active chlorine, such as Cl_2_, HClO, and ClO^−^.

6 2Cl^−^ → Cl_2_ + 2e^−^

7 Cl_2_ + H_2_O → HClO + Cl^−^ + H^+^

8 HClO → ClO^−^ + H^+^

Active chlorine is an essential indirect oxidant in degrading organic matter in electrocatalytic oxidation.^58^ As a result, PVC removal was improved at a pH close to 3.

#### 3.6.7 Effect of anions

**Fig. 16:**
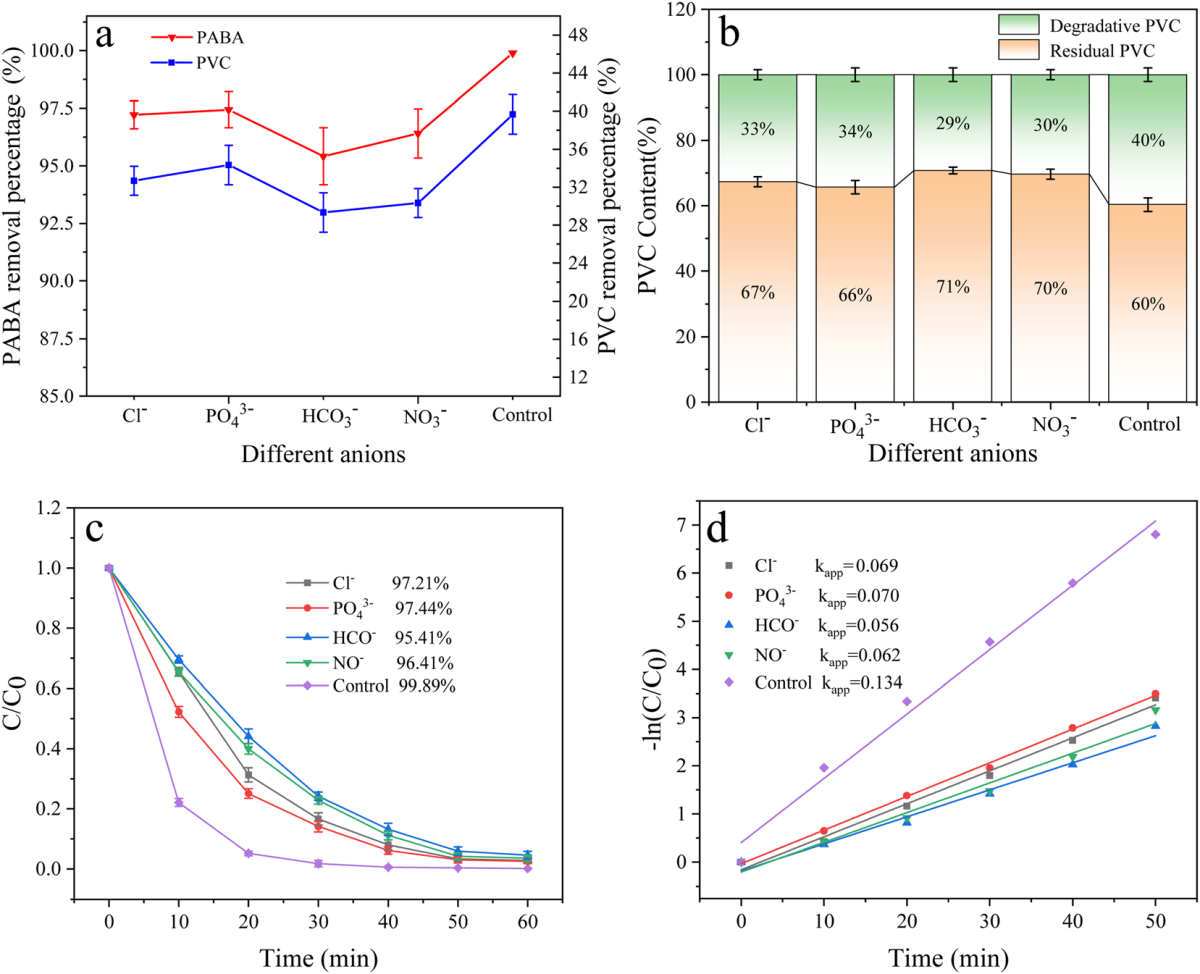
(a) Effect of different anions on PVC and PABA removal percentage. (b) Effect of different anions on PVC degradation. (c) Effect of different anions on PABA degradation. (d) First-order kinetic model of PABA degradation.

Inorganic anions are prevalent in natural water bodies and exert a significant influence on the wastewater treatment process.^59^ The effect of these anions on the degradation of PABA and PVC in the reaction system was investigated by adding 5 mM NaCl, Na_3_PO_4_, NaHCO_3_, and NaNO_3_ to the reaction solution. The result is shown in Fig. 16; the degradation efficiency of PVC and PABA was found to decrease due to the introduction of anions. The observed phenomenon can generally be ascribed to the reaction between the anion and the active substance, resulting in a decrease in the concentration of the active substance within the reaction solution, thereby impacting contaminant removal.^60^ Relevant findings have been documented in other scholarly investigations.^61,62^

### 3.7 Final degradation condition

**Fig. 17:**
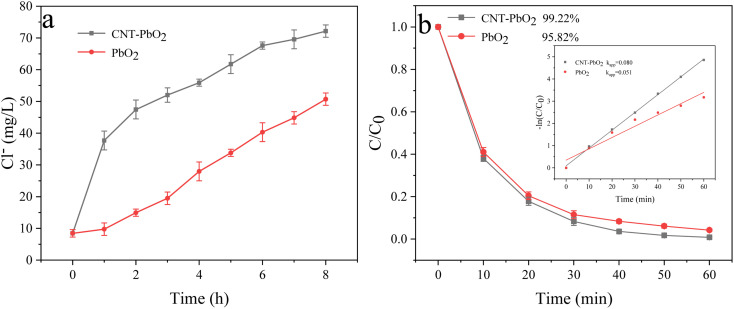
(a) Change in Cl^−^ concentration during PVC degradation. (b) Degradation curve and first-order kinetic model of PABA under the final degradation condition.

The optimal degradation conditions for the simultaneous removal of PABA and PVC were different in the UV/electroactivated persulfate system. At 90 °C, 0.09 g PMS dosage, 0.05 mol L^−1^ electrolyte concentration, 120 mA cm^−2^ current density, and pH 9, PABA removal was optimal, and the degradation efficiency was the fastest. However, the optimum PVC removal was achieved at 90 °C, 0.08 g PMS dosage, 0.05 mol L^−1^ electrolyte concentration, 100 mA cm^−2^ current density, and pH 3. For the excellent co-degradation of PABA and PVC, the final reaction conditions were determined as follows: 90 °C temperature, 0.08 g PMS dosage, 0.05 mol L^−1^ electrolyte concentration, pH 9, and 100 mA cm^−2^ current density. Fig. 17a shows the change in Cl^−^ concentration as different electrodes degrade PVC under the final reaction conditions. When PVC was treated for 8 h with CNT-PbO_2_ and PbO_2_ electrodes, the Cl^−^ concentration was 72.1 mg L^−1^ and 50.7 mg L^−1^, respectively. Fig. 17a shows that the degradation of the CNT-PbO_2_ electrode was fast in the initial 1 hour before slowing down. Moreover, PABA degradation under this condition and the first-order kinetics are illustrated in Fig. 17b. The PABA removal efficiency reached 99.22% and 95.82%, respectively. The degradation effect of the CNT-PbO_2_ electrode was only 0.67% lower than that achieved under the best conditions.

### 3.8 Free radical analysis

**Fig. 18:**
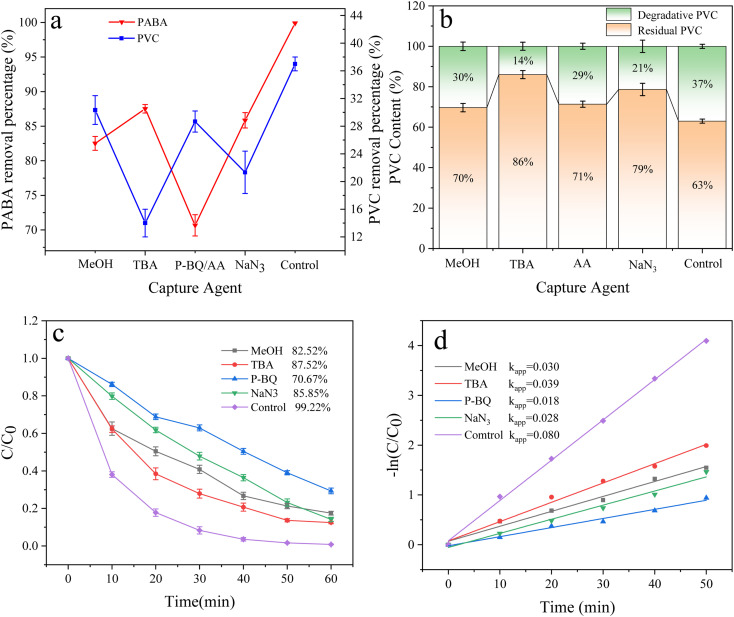
(a) Effect of free radical inhibitors on PVC and PABA removal percentage. (b) Effect of free radical inhibitors on PVC degradation. (c) Effect of free radical inhibitors on PABA degradation. (d) First-order kinetic model of PABA degradation.

Under the optimum conditions, the experimental results of free radical quenching by adding various trapping agents during the degradation of PABA and PVC in the UV/electric co-activation persulfate system are shown in Fig. 18. When TBA was used as the ˙OH collector, the removal rates of PABA and PVC were reduced by 11.7% and 23%, respectively, compared with the control group. Adding MeOH as a common scavenger of SO_4_˙^−^ and ˙OH decreased PABA and PVC degradation by 16.7% and 7%, respectively. This result proves that ˙OH and SO_4_˙^−^ significantly influence PVC degradation.

In exploring the effect of O_2_˙^−^ on PVC degradation, ascorbic acid (AA) was used to inhibit O_2_˙^−^. Since the UV absorption peaks of AA and PABA are highly coincident, P-BQ was selected as the O_2_˙^−^ removal agent to investigate the contribution of O_2_˙^−^ to PABA degradation. We found that PABA and PVC removal decreased by 28.6% and 8.0%, respectively, confirming that O_2_˙^−^ contributed significantly to PABA degradation.

Similarly, NaN_3_ is a ^1^O_2_ trapping agent. After adding NaN_3_, PABA and PVC degradation was reduced by 13.37% and 16%, respectively, indicating that ^1^O_2_ was involved in the degradation of PABA and PVC in the UV/electric co-activated persulfate system.

**Fig. 19:**
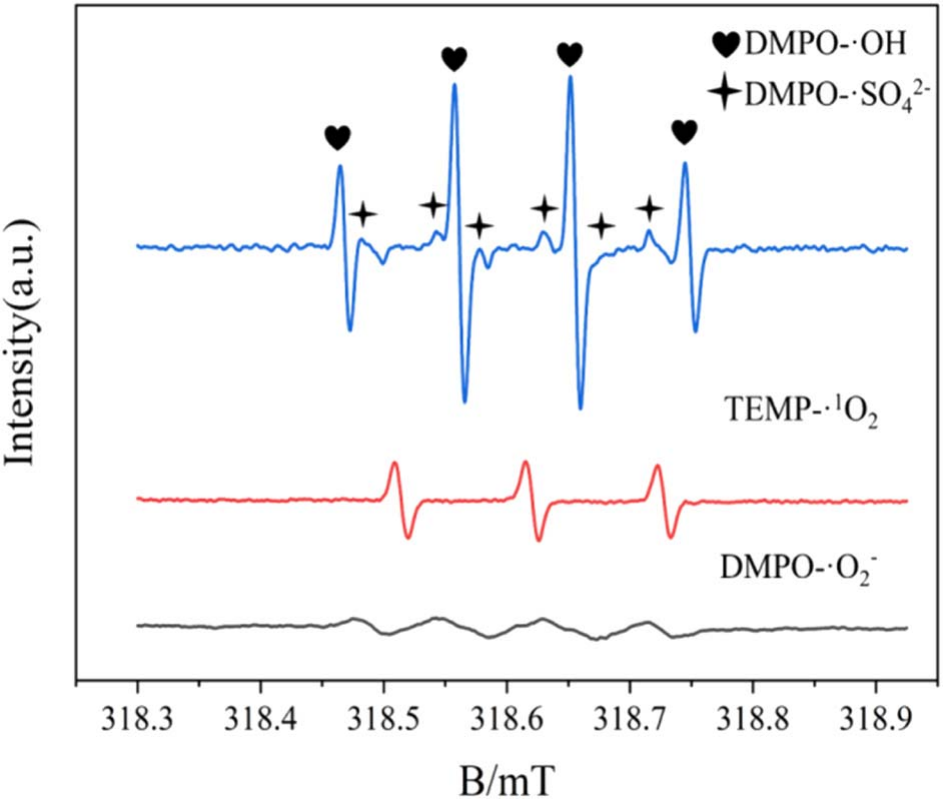
Electron paramagnetic resonance (EPR) signal detected by a spin catcher.

Furthermore, the electron paramagnetic resonance (EPR) explored the active components produced during the experiment. Here, 5,5-dimethyl-1-pyridinane-*N*-oxide (DMPO) was used as a spin trap to identify ˙OH, SO_4_˙^−^, and O_2_˙^−^, while 4-hydroxy-2,2,6,6-tetramethyl-1-piperidine (TEPM) was used to capture ^1^O_2_. As shown in Fig. 19, a typical DMCO–˙OH adduct signal intensity ratio of 1 : 2 : 2 : 1 peak and a six-wire ESR signal of DMPO–SO_4_˙^−^ were detected in the UV/electric co-activated persulfate system. The strong three-wire characteristic peaks of the TEMP–^1^O_2_ adduct with a signal ratio of 1 : 1 : 1 and those of DMPO–O_2_˙^−^ adduct were also detected. These observations are consistent with the results from the capture experiment, indicating that ˙OH, SO_4_˙^−^, O_2_˙^−^, and ^1^O_2_ were actively involved in the degradation.

### 3.9 Degradation mechanism

**Fig. 20:**
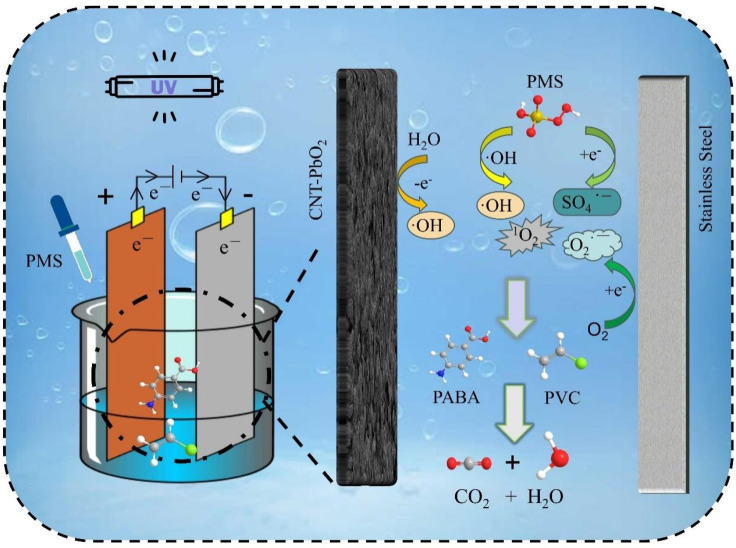
Possible mechanism of ROS production in the activated persulfate system by UV/electric co-promotion.

Fig. 20 illustrates the ROS generation mechanism when PMS was co-activated by UV/electricity to degrade PABA and PVC simultaneously.

(i) The CNT-PbO_2_ anode produces ˙OH *via* water electrolysis. PMS electrocatalytic activation produces SO_4_˙^−^ and ˙OH.

9 M + H_2_O → M(˙OH) + H^+^ + e^−^

10 HSO_5_^−^ + ˙OH → SO_5_˙^−^ + H_2_O

11 2SO_5_˙^−^ → 2SO_4_˙^−^ + O_2_

12 2SO_5_˙^−^ → S_2_O_8_^2−^ + O_2_

13 S_2_O_8_^2−^ + HO_2_˙ → SO_4_˙^−^ + SO_4_^2−^ + O_2_˙^−^ + 2H^+^

14 HSO_5_^−^ + e^−^ → ˙OH + SO_4_^2−^

15 HSO_5_^−^ + e^−^ → SO_4_˙^−^ + OH^−^

(ii) O_2_ is reduced to O_2_˙^−^. PMS is co-activated by UV/electricity to produce O_2_˙^−^.

16 e^−^ + O_2_ → O_2_˙^−^

17 SO_5_˙^−^ + H_2_O → O_2_˙^−^ + SO_4_^2−^ + 2H^+^

(iii) O_2_˙^−^ reacts with ˙OH to produce ^1^O_2_, and PMS also produces ^1^O_2_ through self-decomposition.

18 O_2_˙^−^ + ˙OH → OH^−^ + ^1^O_2_

19 HSO_5_^−^ + SO_5_˙^−^ → HSO_4_^−^ + SO_4_^2−^ + ^1^O_2_

### 3.10 Degradation pathway analysis

#### 3.10.1 PABA degradation pathways

**Fig. 21:**
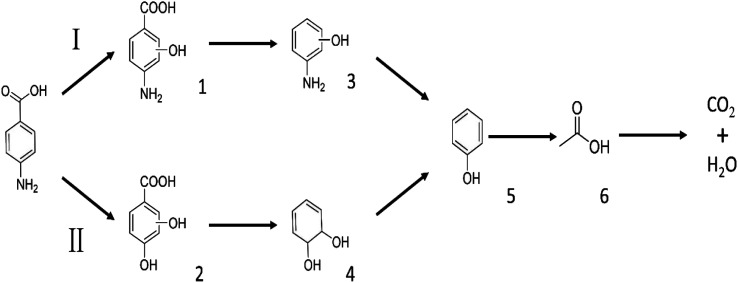
Possible pathways for PABA degradation by UV/electric activation persulfate system.

We employed a liquid chromatography-mass spectrometer to analyze the intermediates generated during PABA degradation to explore and propose the degradation pathways (Fig. 21). In the first pathway, ˙OH induces a PABA substitution reaction to form product 1. Under the action of ˙OH and other ROS, product 1 undergoes a de-carboxyl (de-COOH) reaction to yield product 3. Product 3 is further oxidized to produce product 5, which ROS then attacks to undergo a ring-opening reaction, eventually converting it to CO_2_ and H_2_O. Alternatively, oxidation occurs at the amino position and is accompanied by other substitution reactions, resulting in product 2. The active substance in the solution acts on product 2, causing it to undergo hydroxylation and de-COOH reactions, resulting in derivative 4. Finally, under the attack of multiple ROS, these intermediates are converted into CO_2_ and H_2_O. Table 3 and Fig. S13† show the intermediates that may be produced during PABA degradation in the ultraviolet/electroactivated persulfate system.

**Table 3:** Possible intermediates produced during PABA degradation by the UV/electro activation persulfate system

Sample	Structure	Molecular formula	Molecular mass
1	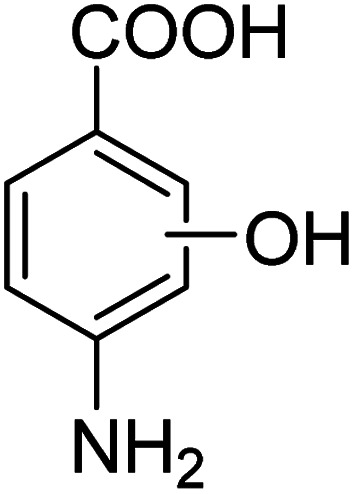	C_8_H_11_NO_3_	169.18
2	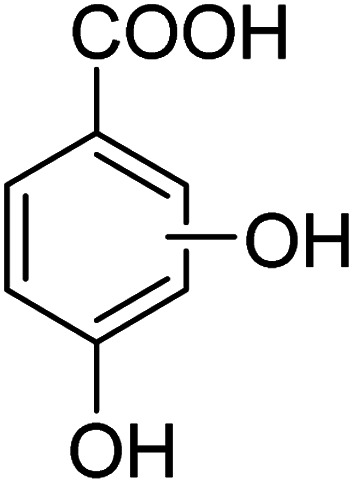	C_8_H_10_O_4_	170.16
3	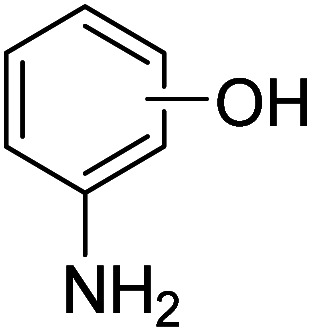	C_7_H_11_NO	125.17
4	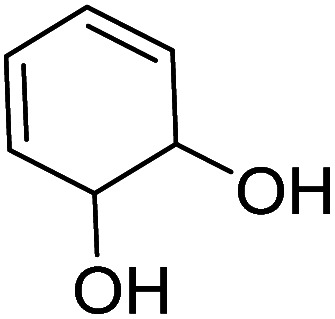	C_6_H_8_O_2_	112.13
5	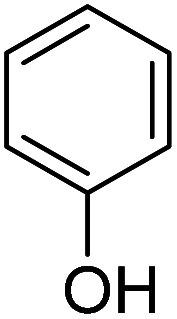	C_6_H_6_O	94.11
6	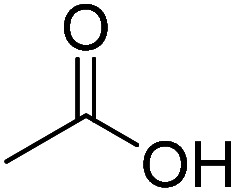	C_2_H_4_O_2_	60.05

#### 3.10.2 PVC degradation pathways

**Fig. 22:**
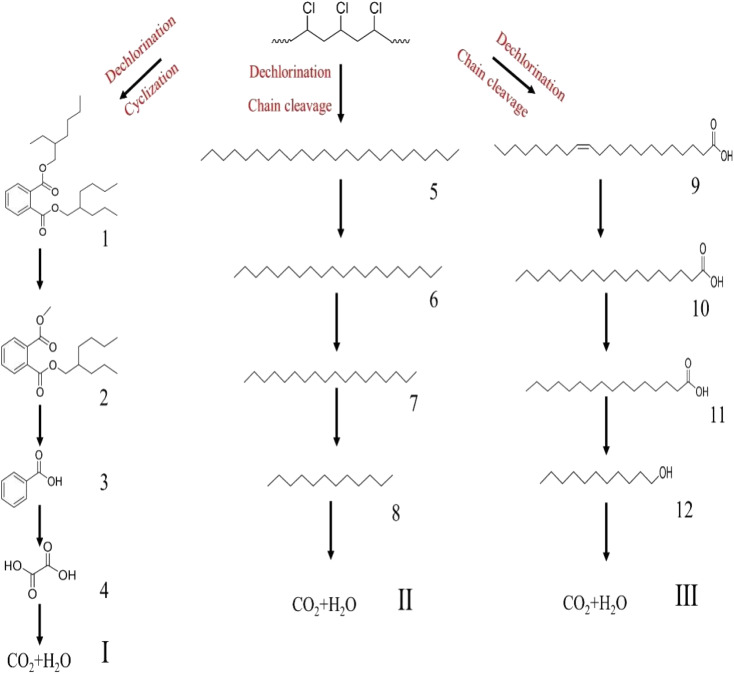
Possible pathways for PVC degradation by UV/electric activation persulfate system.

The degradation pathways of PVC were explored according to the intermediate products observed using LC-MS (Fig. 22). Because the C–Cl bond has the lowest bond energy in the degradation,^63^ it breaks first. After the dechlorination, the reaction could follow two degradation pathways. First, the PVC chain is cracked upon heating and attacked by active components in the solution. Then, a cyclization reaction occurs to form a benzene series. These intermediates are broken down into smaller molecules in the presence of ROS and mineralized into H_2_O and CO_2_ by oxidation. Another pathway is that the PVC chain collapses after cracking to become an organic intermediate in the solution. ROS attacks the shed intermediates to form alcohols, alkanes, and carboxylic acids, eventually oxidized to CO_2_ and H_2_O. The intermediates that may be produced during PVC degradation by the UV/electroactivation persulfate system are provided in Table 4 and Fig. S14.†

**Table 4:** Possible intermediates produced during PVC degradation by UV/electro activation persulfate system

Sample	Structure	Molecular formula	Molecular mass
1	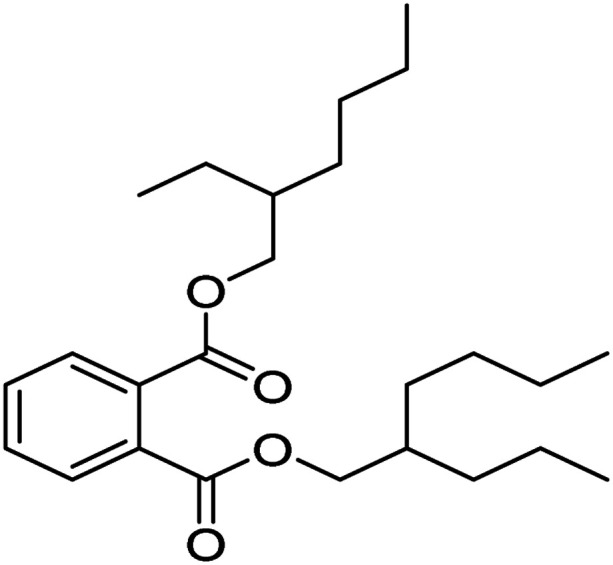	C_25_H_40_O_4_	404.59
2	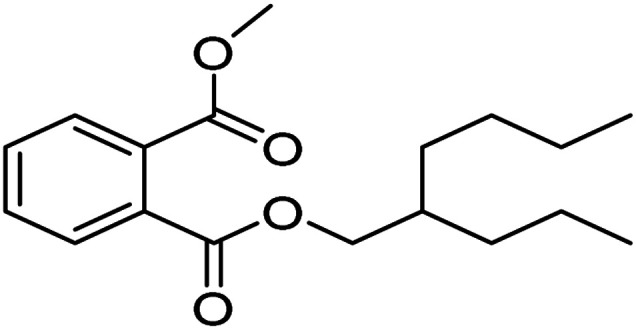	C_18_H_26_O_4_	306.40
3	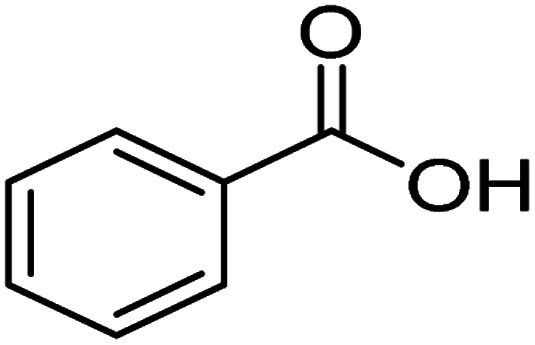	C_7_H_6_O_2_	122.12
4	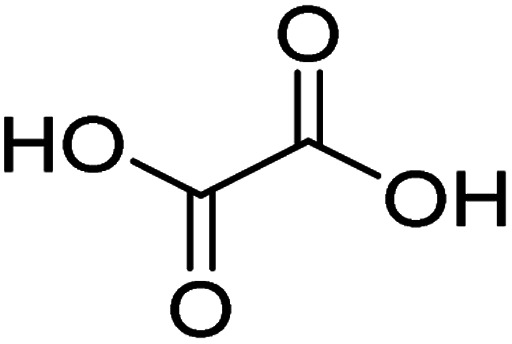	C_2_H_2_O_4_	90.03
5	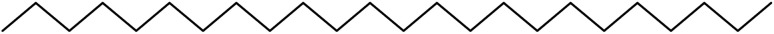	C_24_H_50_	388.66
6	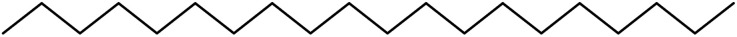	C_20_H_42_	282.56
7	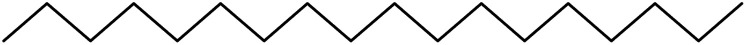	C_18_H_38_	254.30
8	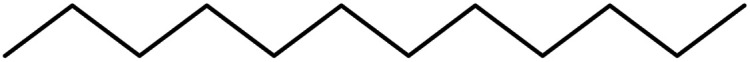	C_12_H_26_	170.34
9	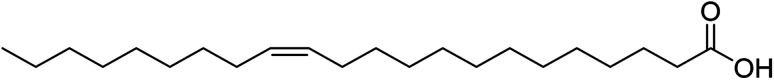	C_22_H_42_O_2_	388.58
10	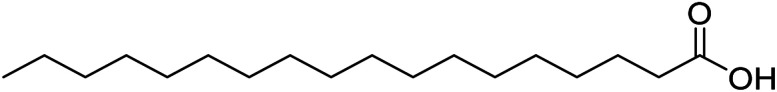	C_18_H_36_O_2_	284.48
11	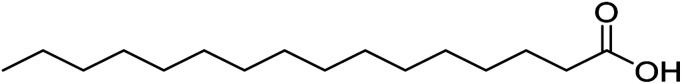	C_16_H_32_O_2_	256.43
12	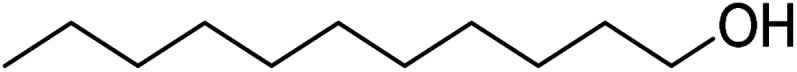	C_11_H_24_O	172.31

#### 3.10.3 Toxicity assessment of intermediates

The toxicity of the intermediate product was assessed using the Toxicity Evaluation Software (TEST), considering parameters such as oral rat LD_50_ and mutagenicity. The oral dose of PABA in rats was 2935.67 mg kg^−1^, as presented in Table S1.† Following the degradation reaction, the LD_50_ value of the final intermediate product exhibited a significant increase, suggesting a reduction in its original.^64,65^ It is worth noting that the toxicity assessment software (TEST) did not yield data for PVC microplastics. The oral LD_50_ values of intermediate products 3 and 4 were determined to be 1262.29 mg kg^−1^ and 1415.78 mg kg^−1^, respectively, indicating high toxicity. However, the LD_50_ value of product 8 exhibited an increase. Nevertheless, it should be emphasized that the majority of intermediate products derived from PVC exhibit a high level of LD_50_ values; specific data are shown in Table S2.† Furthermore, both PABA and PVC intermediates exhibited a lack of mutagenicity. In summary, although certain intermediates exhibit high toxicity, their toxicity diminishes over time due to degradation. Consequently, the response time can be prolonged to mitigate potential risks.

## 4 Conclusion

In this study, we modified the PbO_2_ electrode by CNT doping. We successfully used a UV/electric co-activated persulfate system to achieve aminobenzoic acid (PABA) co-degradation with a typical microplastic polyvinyl chloride (PVC) and organic sunscreen. The surface of the CNT-PbO_2_ electrode was more porous, and the particle size was smaller than that of the PbO_2_ electrode. XPS indicates that CNT-PbO_2_ had a higher O_ads_ load content. The electrochemical characterization showed that CNT doping improved the anode's oxygen evolution potential and specific surface area but lowered the *R*_ct_.

Further, the effect of the UV/electrically co-activated persulfate system on PVC and PABA degradation under various reaction conditions was investigated. Under optimal conditions, PVC and PABA removal was 37% and 99.22%, respectively. The degradation results showed that when the PABA content is high, the pH of the reaction system should be adjusted to optimize the removal, whereas pH 3 is advised when the PVC content is high. Finally, possible PVC and PABA degradation pathways were inferred by detecting the intermediate products using LC-MS technology.

## Conflicts of interest

The authors declare that they have no known competing financial interests or personal relationships that could have appeared to influence the work reported in this paper.

## Supplementary Material

RA-014-D4RA01449A-s001
