# Supplementary material for: Photo-electrochemical activation of persulfate for the simultaneous degradation of microplastics and personal care products
Source: RSC Adv. 2024 May 20;14(23):16150–69. doi: 10.1039/d4ra01449a (PMC11103671; doi:10.1039/d4ra01449a)
Supplement: RA-014-D4RA01449A-s001 [file RA-014-D4RA01449A-s001.pdf]

Electronic Supplementary Material (ESI) for RSC Advances.
This journal is © The Royal Society of Chemistry 2024

Supplementary Material to:

Photo-electrochemical activation of persulfate for simultaneous degradation of microplastics and personal care products

Jiacheng Huang[1], Wanyue Wang[1], Tao Wu[1], Xin Ren[1,2] *, Xuesong Zhao[1,2] **

Total number of pages: 8

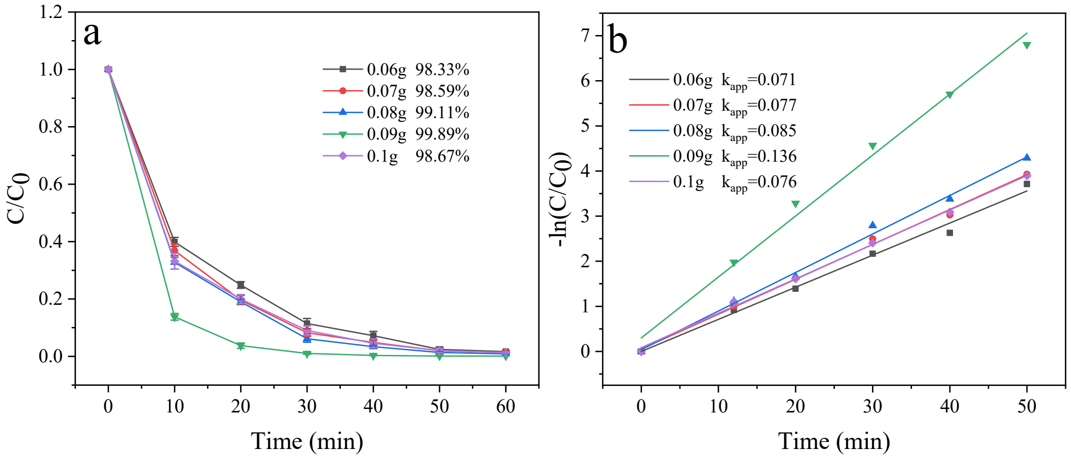

Fig.S1.(a) Effect of PMS dosage on degradation of PABA. (b) First order kinetic line of degradation of PABA by PMS dosage.

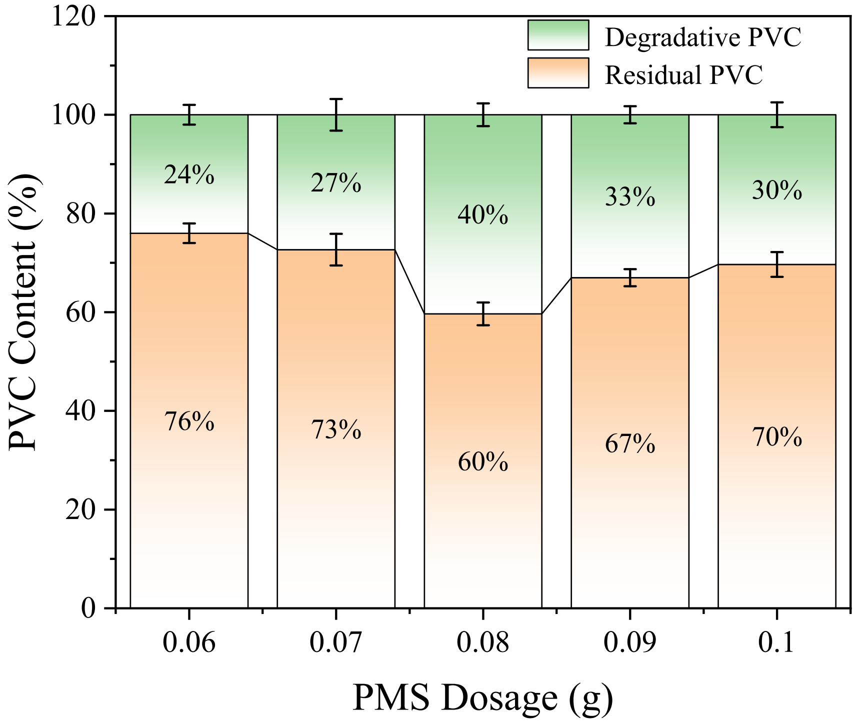

Fig.S2. Effect of PMS dosage on degradation of PVC.

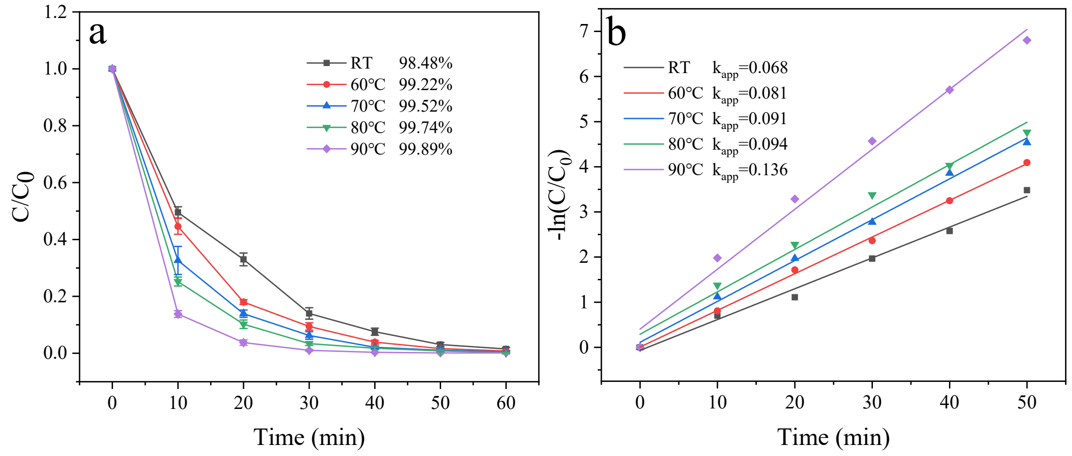

Fig.S3. (a) Effect of temperature on degradation of PABA. (b) First order kinetic line of degradation of PABA by temperature.

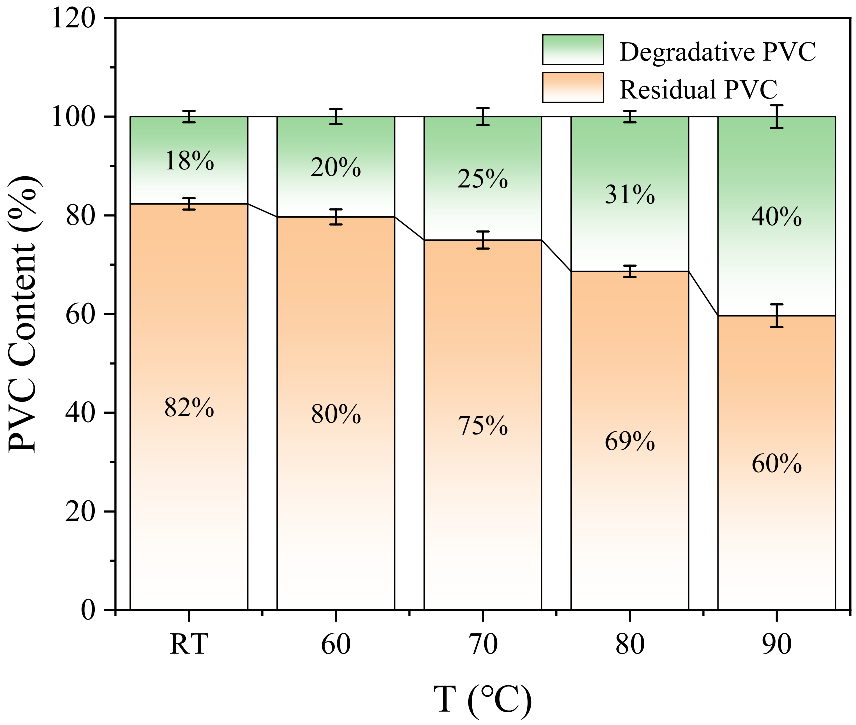

Fig.S4. Effect of temperature on degradation of PVC.

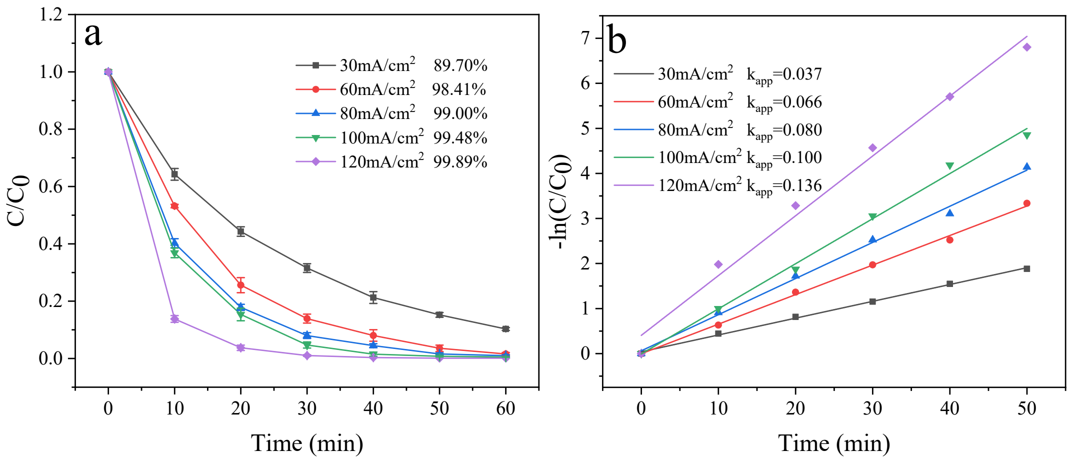

Fig.S5. (a) Effect of current density on degradation of PABA. (b) First order kinetic line of degradation of PABA by current density.

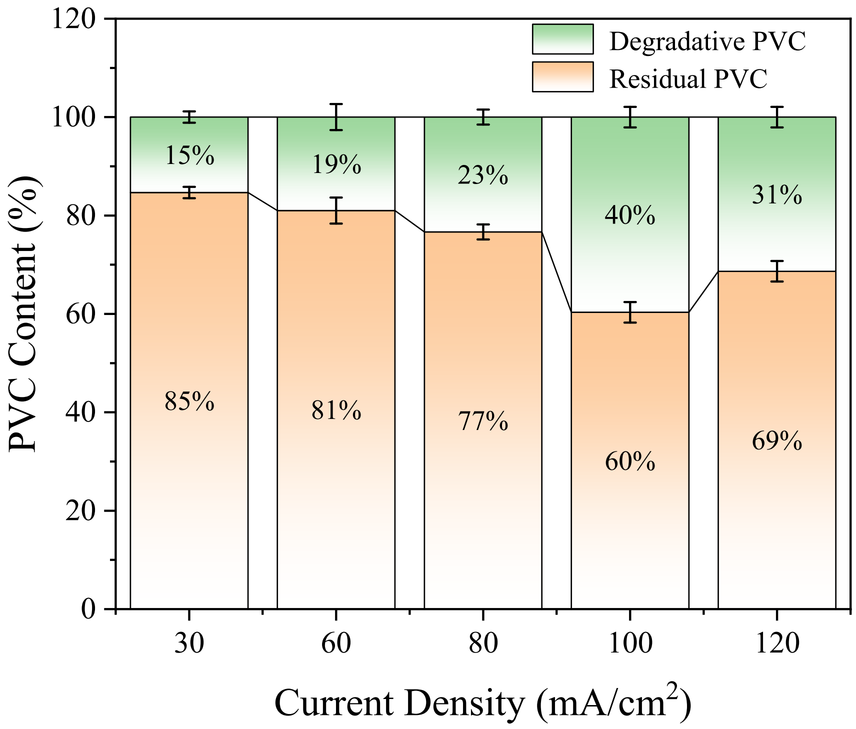

Fig.S6. Effect of current density on degradation of PVC.

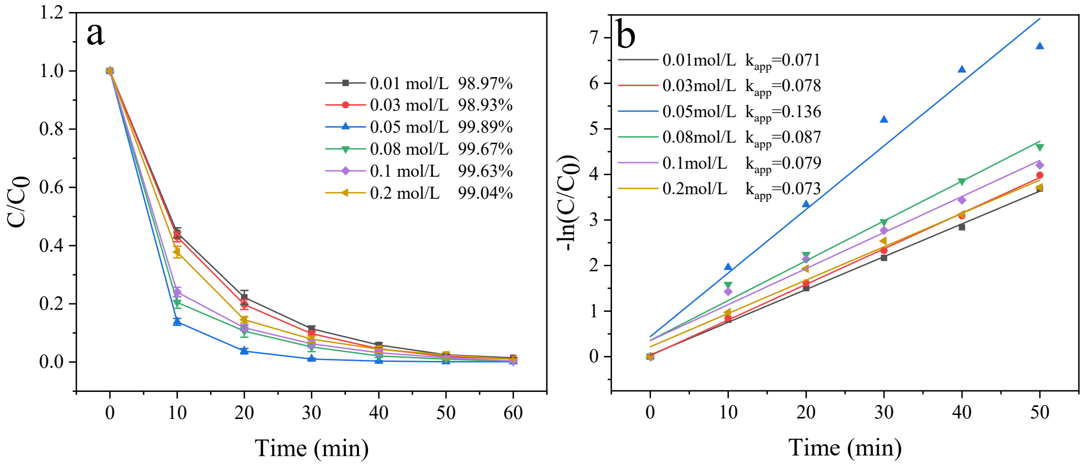

Fig.S7. (a) Effect of electrolyte concentration on degradation of PABA. (b) First order kinetic line of degradation of PABA by electrolyte concentration.

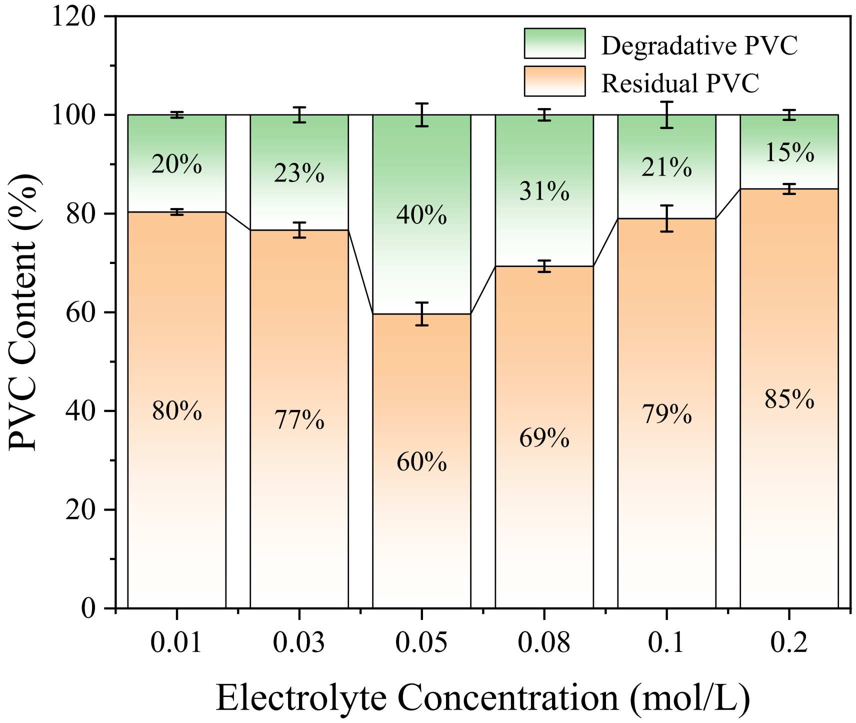

Fig.S8. Effect of electrolyte concentration on degradation of PVC.

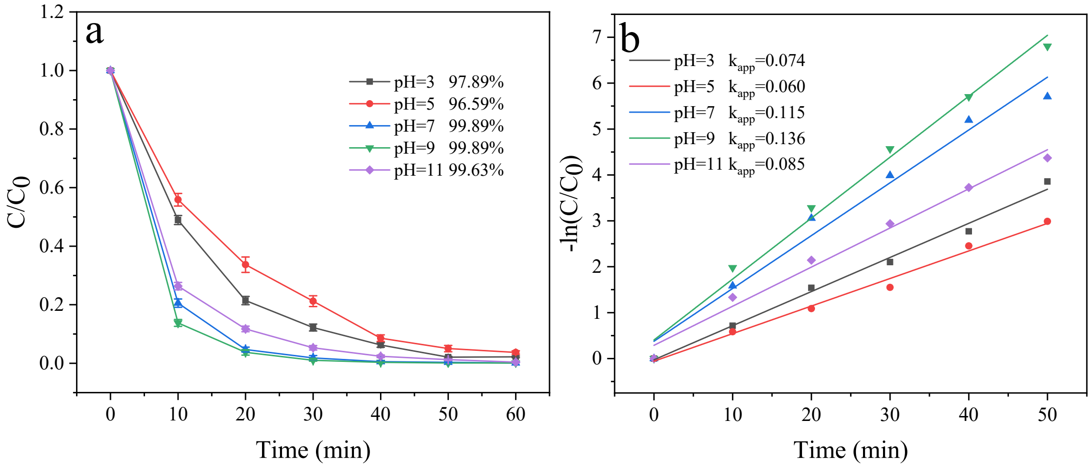

Fig.S9. (a) Effect of pH on degradation of PABA. (b) First order kinetic line of degradation of PABA by pH.

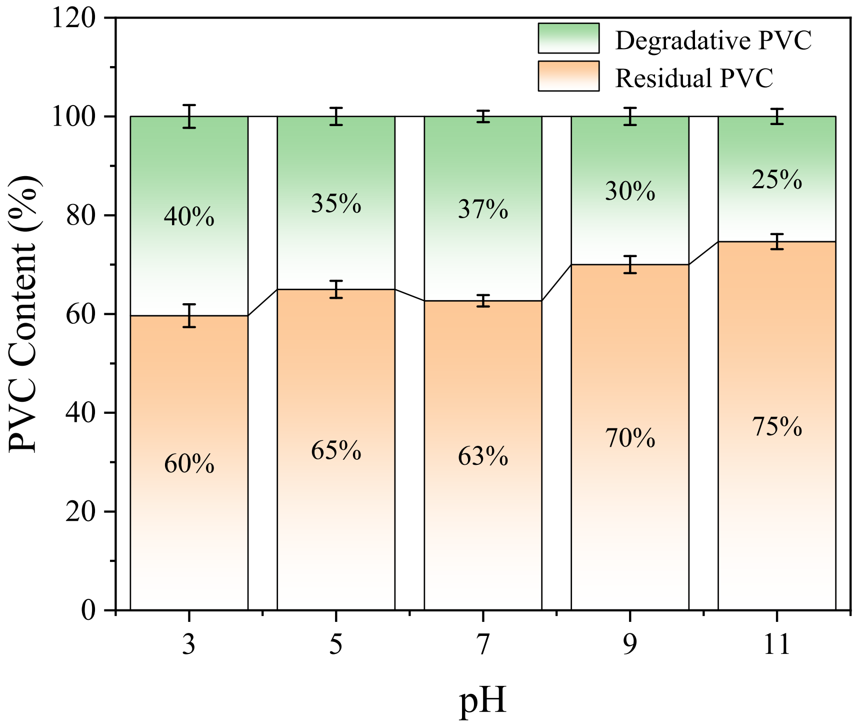

Fig.S10. Effect of pH on degradation of PVC.

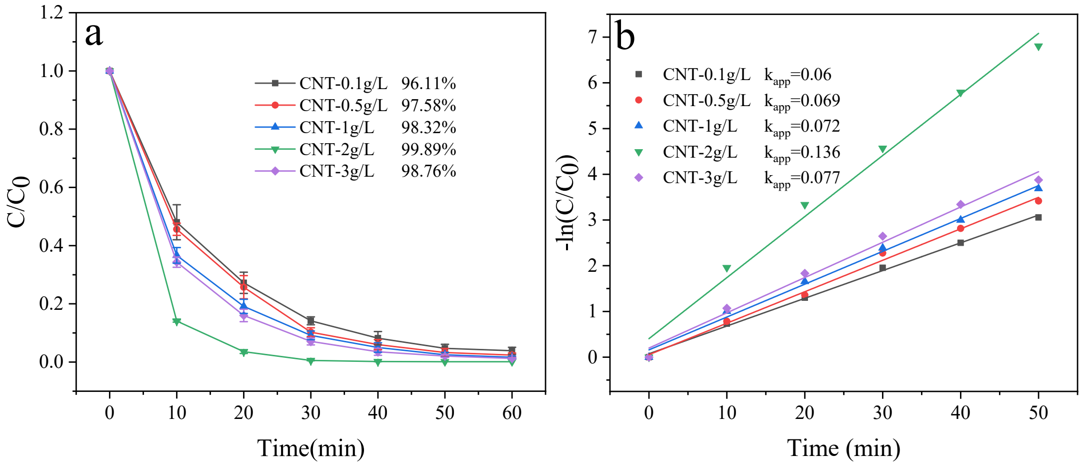

Fig.S11. (a) Effect of CNT dosage on degradation of PABA. (b) First order kinetic line of degradation of PABA by CNT dosage.

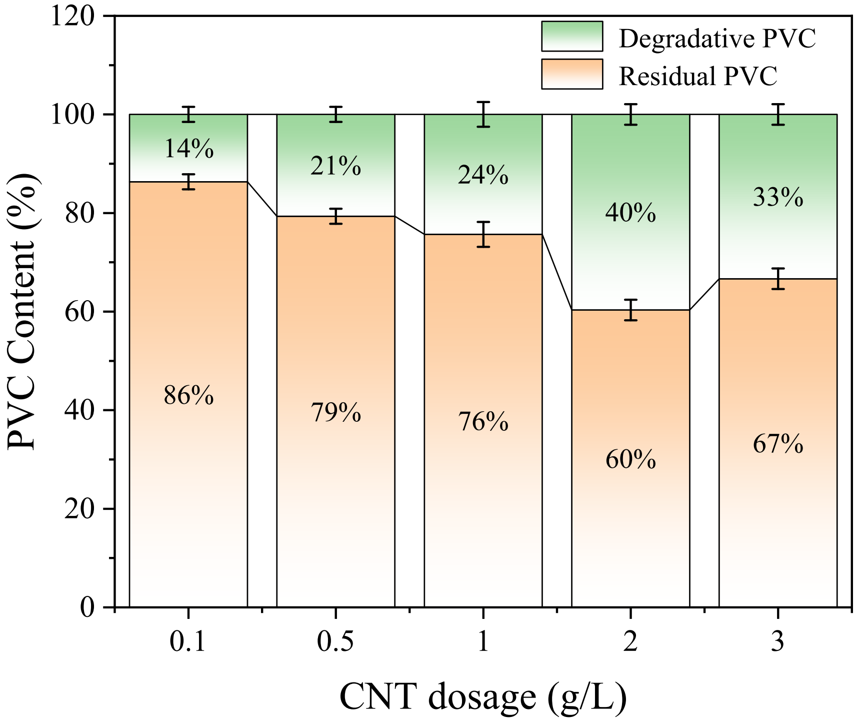

Fig.S12. Effect of CNT dosage on degradation of PVC.

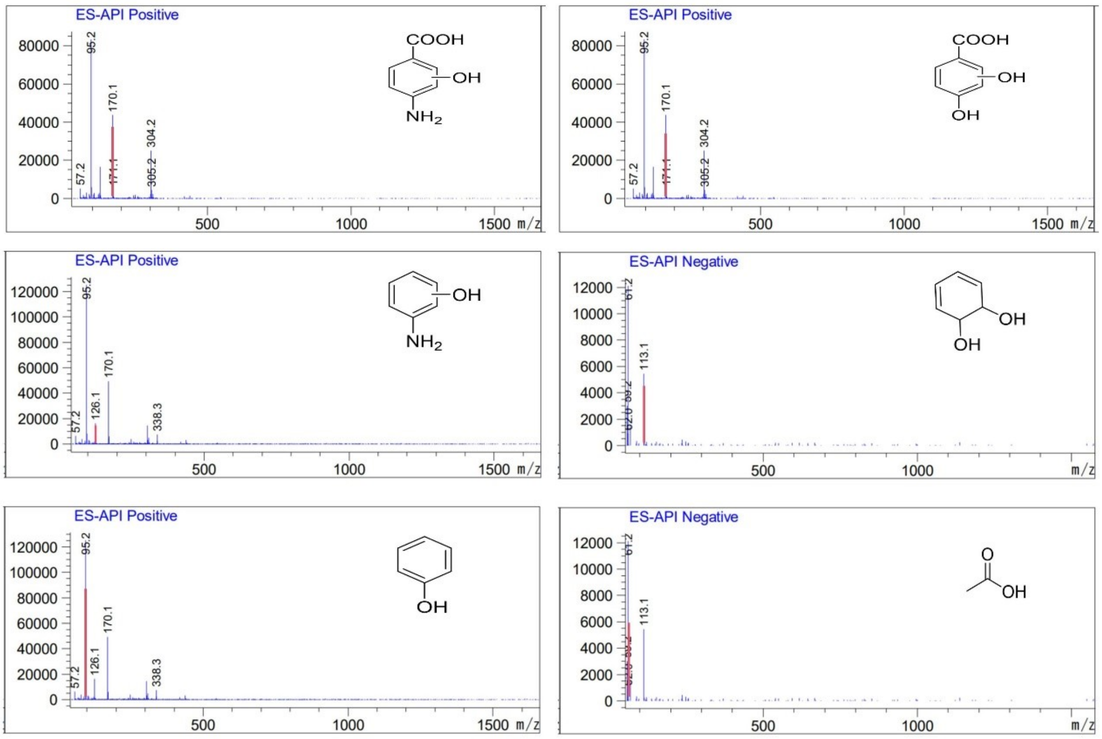

Fig.S13. The LC-MS spectra of degradation intermediates of PABA.

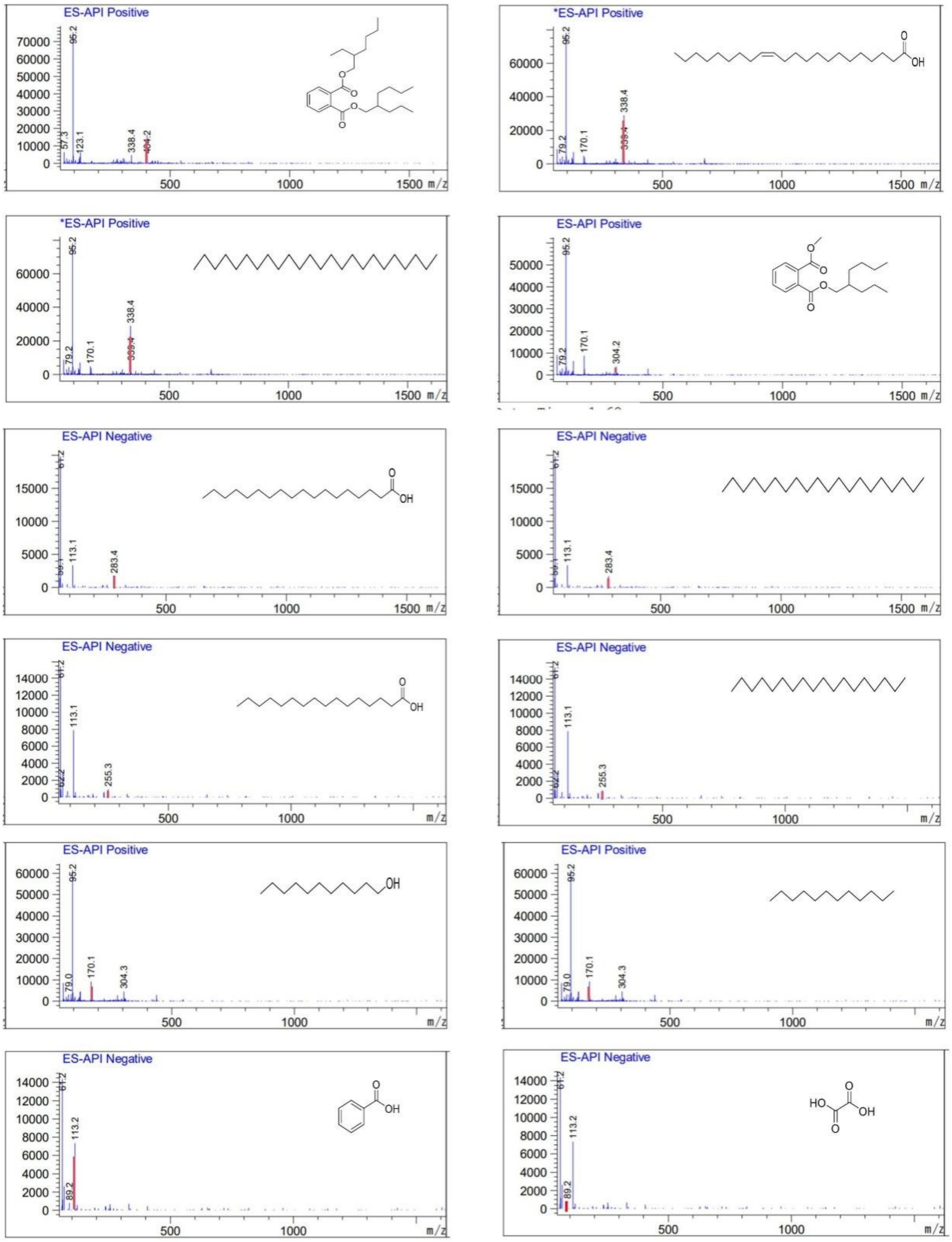

Fig.S14. The LC-MS spectra of degradation intermediates of PVC.

Table S1 Toxicity analysis of PABA intermediates

sample	Structure	Oral rat LD_{50} (mg/Kg)	Mutagenicity
PABA	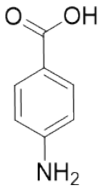	2935.67	0.14 (Negative)
1	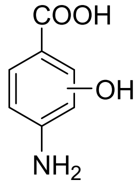	3977.54	0.20 (Negative)
		2630.26	0.13 (Negative)
2	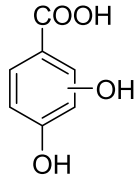	2109.38	0.15 (Negative)
		1621.59	0.19 (Negative)
3	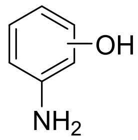	406.69	0.26 (Negative)
		823.36	0.33 (Negative)
4	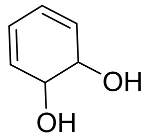	1814.95	0.32 (Negative)
5	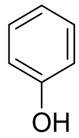	434.02	0.25 (Negative)
6	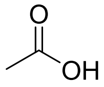	3467.62	-0.06 (Negative)

Note: Due to the identical mass-charge ratio, it is not feasible to ascertain the specific location of hydroxyl groups in products 1, 2, and 3; hence, both toxicities are indicated in the table S1.

Table S2 Toxicity analysis of PVC intermediates

sample	Structure	Oral rat LD_{50} (mg/Kg)	Mutagenicity
1	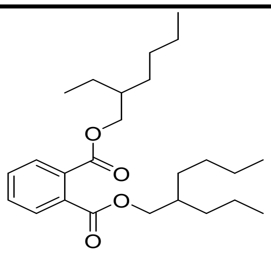	27204.16	0.04 (Negative)
2	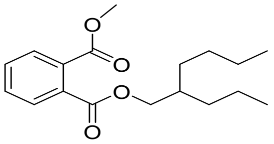	17345.67	0.06 (Negative)
3	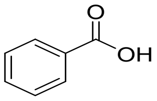	1262.29	-0.02 (Negative)
4	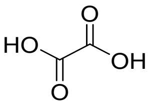	1415.78	0.30 (Negative)
5	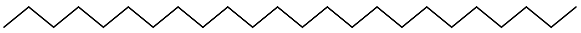	8842.74	-0.09 (Negative)
6	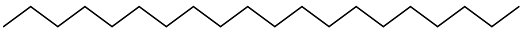	4607.09	-0.06 (Negative)
7	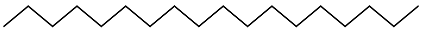	5686.06	-0.07 (Negative)
8	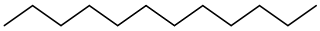	5855.12	-0.14 (Negative)
9	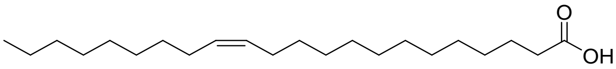	16527.98	0.01 (Negative)
10	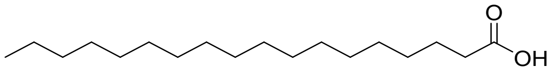	12645.29	-0.07 (Negative)
11	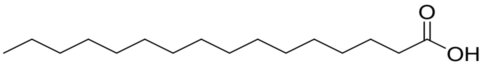	10881.96	-0.09 (Negative)
12	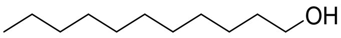	5480.48	-0.01 (Negative)
